# RELACS nuclei barcoding enables high-throughput ChIP-seq

**DOI:** 10.1038/s42003-018-0219-z

**Published:** 2018-12-05

**Authors:** Laura Arrigoni, Hoor Al-Hasani, Fidel Ramírez, Ilaria Panzeri, Devon Patrick Ryan, Diana Santacruz, Nadia Kress, John Andrew Pospisilik, Ulrike Bönisch, Thomas Manke

**Affiliations:** Max Planck Institute of Immunobiology and Epigenetics, Stübeweg 51, 79108 Freiburg, Germany

**Keywords:** Chromatin analysis, Epigenomics

## Abstract

Chromatin immunoprecipitation followed by deep sequencing (ChIP-seq) is an invaluable tool for mapping chromatin-associated proteins. Current barcoding strategies aim to improve assay throughput and scalability but intense sample handling and lack of standardization over cell types, cell numbers and epitopes hinder wide-spread use in the field. Here, we present a barcoding method to enable high-throughput ChIP-seq using common molecular biology techniques. The method, called RELACS (restriction enzyme-based labeling of chromatin in situ) relies on standardized nuclei extraction from any source and employs chromatin cutting and barcoding within intact nuclei. Barcoded nuclei are pooled and processed within the same ChIP reaction, for maximal comparability and workload reduction. The innovative barcoding concept is particularly user-friendly and suitable for implementation to standardized large-scale clinical studies and scarce samples. Aiming to maximize universality and scalability, RELACS can generate ChIP-seq libraries for transcription factors and histone modifications from hundreds of samples within three days.

## Abstract (web summary)

Laura Arrigoni et al. present RELACS, a method enabling high-throughput ChIP-seq which involves barcoding and processing intact nuclei in the same ChIP reaction. The method is useful for broad cell types and epitopes, robust to experimental conditions, and drastically decreases workload.

## Introduction

Chromatin immunoprecipitation followed by deep sequencing (ChIP-seq) is a key technique for exploring the genomic location of bound proteins and histone modifications, which has significantly contributed to our understanding of gene regulation and epigenetic changes in healthy and diseased cells.

Recent years have seen a number of methodological advances^1^ to reduce the input requirements for ChIP-seq from millions to few thousands of cells per assay, by using improved sample preparation^2–6^, more sensitive library preparation^4,7,8^, and microfluidic devices^9,10^. Despite these improvements, ChIP-seq still suffers from limitations imposed by the complexity of the protocol, lack of standardization across cell types and epitopes, technical variability, low efficiency for proteins weakly bound to chromatin, and limited throughput. To enhance throughput and facilitate large-scale sample screening, automated immunoprecipitation and library preparation solutions have been proposed^11,12^. More recent ChIP-seq methods^2,3,13^ aim to increase throughput by barcoding the starting chromatin and multiplex several samples within a single ChIP reaction. The multiplexing of samples in the same ChIP dramatically increases the throughput and minimizes technical variability. After immunoprecipitation, barcoded DNA is purified and converted into a sequencing library using a single PCR reaction. Barcode sequences ligated to each starting sample are used to identify signals from each cell population during data analysis.

Supplementary Table 1 shows a comparison between current high-throughput ChIP-seq protocols involving chromatin barcoding prior ChIP^2,3,13–15^ or automation^11,12,16^. Two common strategies have been developed for molecular barcoding of sonicated or enzymatically digested chromatin. The first approach (used in^2,14,15^) relies on barcoding of chromatin immobilized to beads. Two successive chromatin immunoprecipitations are applied in these protocols: after the first immunoprecipitation required for the ligation of barcodes, chromatins are released from the beads and pooled for the second multiplexed ChIP against the epitope of interest. The first immunoprecipitation step performed on each individual sample severely limits assay throughput and does not significantly reduce the workload compared to standard ChIP-seq. Moreover, the repeated ChIP (re-ChIP) step involved may reduce the available material needed for the second ChIP, especially if the efficiency of the first chosen antibody is poor.

The second barcoding strategy used in enzymatic approaches such as Bar-ChIP^13^ and Mint-ChIP^3^, circumvent the need of a re-ChIP step by releasing nucleosomes in solution and directly ligate adapters to chromatin. Still, these protocols lack universal applicability since Bar-ChIP requires millions of cells and Mint-ChIP is exclusively conducted on fresh un-fixed material, impeding the application to proteins weakly bound to chromatin such as transcription factors. Also, storage and transport are logistically more complicated with fresh material. In addition, all these protocols employ MNase to digest chromatin. Although MNase is useful for chromatin footprinting and fine mapping of binding sites because of exonuclease activity^15,17^, it is concentration-sensitive^18,19^ and typically requires pilot experiments to carefully calibrate the enzyme amount when working with different cell types or strains^3,15^, different transcription factors^17^, MNase batches^15^, and may also require the generation of independent datasets of samples digested at low and high MNase concentrations^3^. Therefore, usage of MNase will require experiment-dependent optimizations, hindering standardized sample processing and screenings of rare patient samples that do not allow for protocol optimizations.

To address the shortcomings of current multiplexed ChIP-seq techniques we introduce a barcoding concept to facilitate standardized high-throughput ChIP-seq. The method, called RELACS (restriction enzyme-based labeling of chromatin in situ), relies on standardized nuclei extraction by sonication^5^ followed by intranuclear enzymatic chromatin cutting and barcode ligation. Once multiple cell populations are barcoded, they are pooled for parallelized ChIP-seq. RELACS can be used for a broad spectrum of cell types and epitopes (active and repressive histone modifications, transcription factors, co-factors) and it is robust to variation of cell numbers and experimental conditions. The simple intranuclear barcoding strategy drastically decreases workload and it is ideally suited to adapt innovative combinatorial barcoding concepts^20–22^ currently used for single cell ChIP-Seq analysis. As with other barcoding strategies^3,14,15^, this approach can be used for quantitative ChIP-seq analyses.

## Results

### Method description

**Fig. 1:**
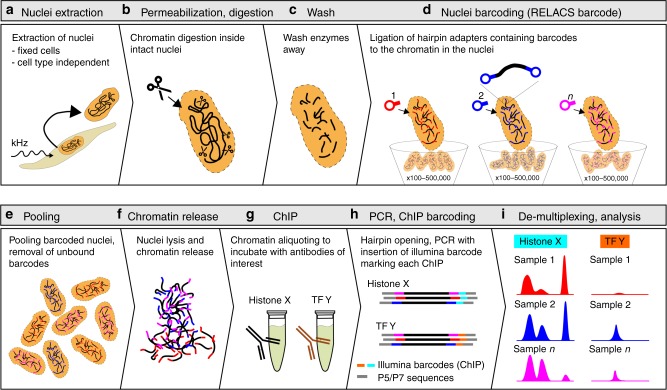
RELACS workflow. Overview of the RELACS method. The protocol facilitates barcoding multiple cell populations, which can be pooled and investigated for multiple epitopes within the same run. The method starts by isolating nuclei from a pool of formaldehyde-fixed cells, using sonication to reduce cell type dependency^5^ (**a**). The nuclear membrane is permeabilized to allow entrance of enzymes, followed by DNA barcodes. Restriction endonucleases with a high frequency of recognition sites are used to fragment chromatin—in this work CviKI-1 was used (**b**). Nuclei are washed to remove active restriction enzymes (**c**). Hairpin adapters harboring barcodes are ligated to both the ends of the fragmented chromatin inside the nuclei. The barcoding has been tested using 100–500,000 nuclei without the need to change protocol conditions (**d**). Cell populations marked with specific barcodes are pooled (**e**), concentrated and lysed using SDS at low concentration and short sonication to release chromatin into solution (**f**). Chromatin is split and incubated with the antibodies of interest (**g**). After ChIP washes and DNA purification (not illustrated), only DNA that harbors nuclei barcodes at both ends is PCR amplified to complete library construction. PCR amplification appends an Illumina barcode onto mark each fragment (**h**). Sequenced libraries are demultiplexed by Illumina barcode, to retrieve ChIP information, and then nuclear barcode, to identify the initial cell population (**i**). The RELACS protocol is very fast and ChIP-seq libraries can be generated for hundreds of samples within three days

RELACS includes several steps to integrate DNA barcodes on chromatin prior to immunoprecipitation (Fig. 1). Further details are available in the Methods section below and through Protocol Exchange^23^. The key of the protocol is to work with intact nuclei that can be simply precipitated to efficiently wash and remove the different master mixes during the chromatin fragmentation and barcoding process and to reduce volumes after pooling of many barcoded samples. Digestion and ligation conditions are optimized to favor the entrance of the DNA barcodes into the nuclei and their ligation to fragmented chromatin while minimizing chromatin self-ligation.

The protocol starts by isolating clean nuclei from fixed cells using sonication, which has been previously demonstrated to outperform state-of-the-art techniques to isolate nuclei from fixed cells and it is a key step to achieve protocol standardization across cell types^5^. Working with well-isolated nuclei makes the following steps of nuclei permeabilization, digestion and in-nuclei barcoding more reproducible.

Nuclei are permeabilized and chromatin is digested by restriction enzymes using a modification^24^ of the procedure used to perform in situ Hi-C^25^. Unlike exonucleases, even large concentrations of restriction enzymes do not risk overcutting chromatin because of their sequence-specificity. This makes the chromatin fragmentation step more robust and predictable.

In this study, the enzyme CvIKI-1 was chosen for several reasons: First it cuts frequently owing to a variable restriction site (RG|CY). Because of the GC motif, it has increased cutting frequency in promoter regions, where many sharp chromatin marks can be resolved. At the same time, this enzyme is insensitive to DNA methylation, which is beneficial when comparing samples with different DNA methylation levels. Importantly, CviKI-1 produces blunt ends which reduces the chance of chromatin re-ligation. We have found that, unlike other tested enzymes, CviKI-1 works well already at room temperature, reducing the risk of crosslink reversal which can happen at higher temperatures^26^.

After digestion, nuclei are precipitated and enzymes are washed away. To increase ligation efficiency, chromatin is A-tailed and customized adapters containing nuclear barcodes are ligated to both ends of the chromatin fragments (Supplementary Fig. 1). This conventional adapter ligation strategy is both robust to variation of cell number (without changing conditions we typically barcode 100–500,000 cells) and practical to implement in various laboratory and clinical settings. Lastly, chromatin containing different barcoded samples can be aliquoted prior to ChIP for the investigation of multiple epitopes. A second barcode is inserted via a PCR step to mark different ChIP experiments or controls. In addition to a simplified workflow and enhanced comparability, the multiplexing of low cell number samples also allows easier handling and reduces potential loss of chromatin during immunoprecipitation.

Many critical steps of the workflow can be quality-controlled in real-time without the need for iterative optimizations. For samples with more than 10,000 cells all checkpoints can be assessed visually or even quantitatively (nuclei extraction, cell number before and after digestion, ChIP and library efficiency), while for samples with lower cell number, successful preparation can be assessed from the final library.

### RELACS fragmentation covers the whole genome with high quality

First we tested that all barcodes are properly included. Their distribution is consistent across epitopes (Supplementary Fig. 2a) and there is no apparent cross-barcode contamination (Supplementary Fig. 2b). The overall mapping rates are very high (> 99%) and we observed only small fractions (3–5%) of disconcordantly and multi-mapping reads (Supplementary Data 1, Supplementary Fig. 2c). These reads were excluded from subsequent analyses.

The RELACS fragmentation strategy does not appreciably affect genomic coverage, as indicated by direct comparison with the traditional method (Supplementary Fig. 3). The only exceptions are heterochromatic regions, where coverage in RELACS data is lower at the same sequencing depth. Importantly, open regions of prime experimental interest are better covered at lower depths. A more detailed analysis of restriction site occurrence in the genome (cutability) and restriction site preference (accessibility) indicates that both are reduced in heterochromatic regions (Supplementary Fig. 4). Other repressed regions are comparable between RELACS and the traditional protocol, while open chromatin regions show higher accessibility for RELACS. As with all ChIP-seq studies, we account for such biases by employing an input control for normalization and peak calling.

### RELACS results are comparable to traditional ChIP-seq

We validated RELACS using a well-studied human cell line (HepG2) for which many external references are available. Here, we generated six histone modification maps for IHEC class I epigenomes (H3K27ac, H3K4me3, H3K4me1, H3K36me3, H3K9me3, H3K27me3), the insulator protein CTCF, as well as co-factor p300. All maps were created using both a traditional sonication-based ChIP-seq protocol (with 100,000 cells per ChIP) and our new RELACS method with 20 technical replicates (20 barcodes × 5000 cells). We also make use of external ENCODE data that was produced with an access of more than a 1,000,000 cells per ChIP (20 M for transcription factors).

**Fig. 2:**
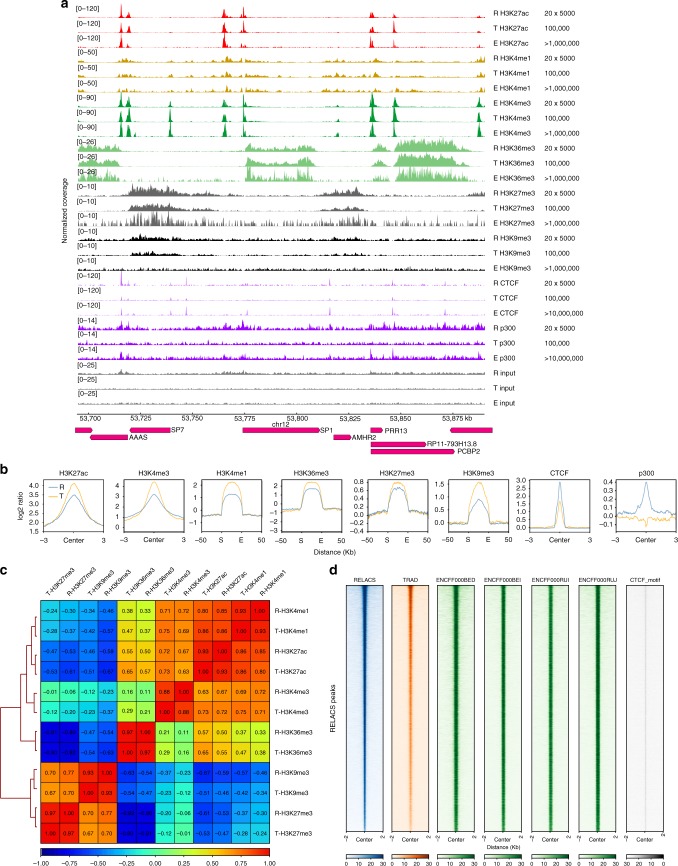
RELACS validation. Using the HepG2 cell line as a model system, we compare results from RELACS with those from traditional ChIP-seq methods. **a** Data tracks for 6 histone marks (H3K4me1, H3K27ac, H3K4me3, H3K36me3, H3K27me3, H3K9me3), transcription factor (CTCF), a co-factor (p300) and Input are shown. Marks prepared with RELACS, designated with an *R*, and a traditional method^5^ are designated with letter *T*. External reference tracks from the ENCODE project are denoted by letter *E*. For RELACS each track show the merged data from 20 × 5000 cells; for traditional protocol each ChIP track corresponds to 100,000 cells, while ENCODE used cell numbers ranging from 1 to 20 million cells. One percent of the chromatin was used to prepare Input samples. **b** Aggregated signal of RELACS and traditional ChIP are shown over peaks from ENCODE (see online methods). For sharp marks, we centered the profile around the annotated peak center and added 3 kb to either side. For broad marks, we scaled the regions to 50 kb and added a flanking region of 50 kb. **c** Pearson correlation of log2-ratios (ChIP/Input) for RELACS (R) and Traditional (T) samples. The correlation was computed for 10 kb bins. **d** Heatmap shows the the signal distributions around 16,092 CTCF peaks from RELACS data and various other references (RELACS: merged signal from 20 × 5000 cells, Traditional: 1 × 100,000 cells); ENCODE data (ENCFF000BED, ENCFF000BEI, ENCFF000RUI, ENCFF000RUJ: > 10 million cells). Additional heatmaps of other marks are shown in Supplementary Fig. 7

All marks show very high quality and reproducibility, as illustrated in Fig. 2a and Supplementary Fig. 5. This can be quantified by quality metrics such as mapping rates, duplication rates and the fraction of reads in annotated peak regions from the ENCODE consortium (see also Supplementary Data 1–2 and Supplementary Fig. 6a). Average profiles around known peaks show excellent agreement of the RELACS method with the traditional approach (Fig. 2b). Notice that the resolution achieved with CviKI-1 is comparable to sonication-based fragmentation, indicating that our choice of restriction enzyme faithfully conveys the underlying signal distributions including transcription factors and co-factors, such as CTCF and p300. In those cases the traditional approach is much poorer or fails when using small cell amounts. Such success cannot be claimed by other multiplexed ChIP-seq protocols^3^, which cannot be applied to transcription factors and also reportedly have problems in mapping sharp histone marks. While the signal of H3K9me3 is reduced because of preferences for open chromatin, RELACS can still resolve the characteristic enrichment over repetitive elements and large heterochromatic domains (Supplementary Fig. 6b). The genome-wide correlations between the log2-ratios (ChIP/Input) from the traditional method and RELACS are high (0.88–0.97, Fig. 2c). We conducted a standard peak calling analysis (using MACS2^27^ and histoneHMM^28^) to compare ChIP-seq signals from various studies over enriched regions and observe good agreement around peak regions called from either RELACS data or defined by ENCODE (Fig. 2d and Supplementary Fig. 7). Specifically for CTCF, there is strong additional support from the expected sequence motif which can be found in 97.4% of all peaks. Unsurprisingly, de-novo motif detection with MEME^29^ for the top 500 RELACS and ENCODE peaks also revealed the same CTCF motif, with E-values 3.3e−752 and 3.4e−714 respectively.

In the case of p300 we observe a good overall concordance of genome-wide signals with ENCODE (Supplementary Fig. 7b), but our stringent peak calling only identified a small number of peaks with significant enrichment over input control (q < 0.05). A much larger number of enriched regions was defined by ENCODE based on a comparison against rabbit-IgG control. In contrast to CTCF, we do not expect a single motif for co-factor p300 which can interact with multiple diverse DNA-binding factors. In our analysis, p300 peaks show generic preferences for GC rich regions (E = 4.6E−55) and might be targets for a large number of C2H2 zinc fingers (such as Sp1).

### Restriction site depletions have only small effects on ChIP

An analysis of the distribution of distances between restriction sites shows that 6088 regions in the human genome, hs37d5, have gaps larger than 1000 bp (Supplementary Fig. 8a). The mouse genome, mm10, has 3695 restriction site gaps larger than 1000 bp. Sequence fragments derived from such gaps (or longer) would not be observable with current Illumina sequencing technology, and any ChIP-seq signal in such gaps will not be detectable. Therefore the RELACS method is blind to 0.29% and 0.18% of the human and mouse genome, respectively. In practice, long gaps tend to occur in heterochromatin and repetitive genome regions (Supplementary Fig. 8b). As might be expected, some H3K9me3 enriched regions are lost in localized restriction gaps, but ChIP-seq signals from repressed chromatin usually spread much larger regions (Supplementary Fig. 8d). This requires more care and robust algorithms to detect larger domains that span intermittent gaps of low mappability. Notice that this is a common problem for all signal analyses in heterochromatin. For sharp marks only a very few regions are affected by restriction site depletion, as illustrated for CTCF (Supplementary Fig. 8c) and summarized for all marks of this study (Supplementary Fig. 8d).

### RELACS works reliably with low cell numbers

**Fig. 3:**
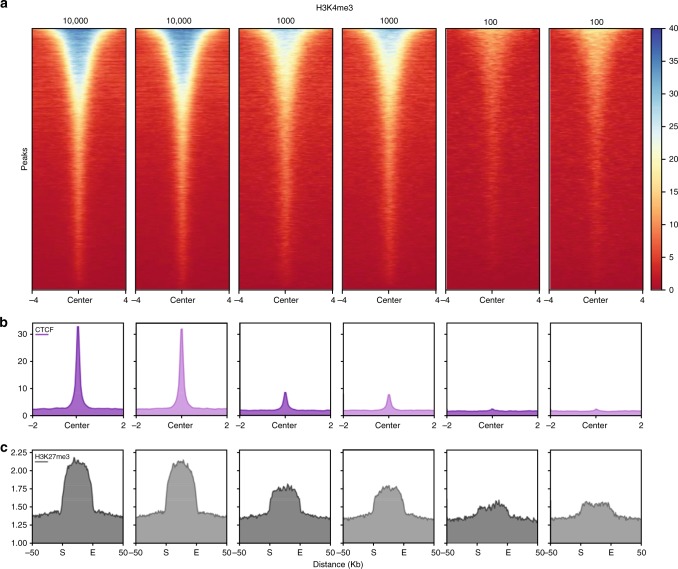
RELACS sensitivity using low cell numbers. Comparison of RELACS results using 10,000, 1000 and 100 HepG2 cells as starting material. For each cell number, 7 technical replicates were barcoded and pooled for ChIP-seq and computationally demultiplexed before analysis. This figure shows two selected replicates from two individual barcodes for each cell number. The top heatmap (**a**) shows the 1×-normalized read coverage for the H3K4me3 histone mark at its respective ENCODE peaks. The coverage is shown centered at the peak with 4 kb flanks on each side. Similarly, for CTCF (**b**) the normalized coverage at the peak center shown together with 2 kb flanking regions. For H3K27me3 (**c**) normalized coverage at each ENCODE peak is scaled to 50 kb (S: start position; E: end position) and flanking 50 kb are shown

Barcoding of chromatin before any further treatment facilitates pooling of scarce samples prior to ChIP, as previously shown^2,3^. Such pooling results in sufficient material for immunoprecipitation and quality controlling, which is typically only possible given samples with abundant material. Here we tested the sensitivity limits of RELACS by barcoding seven replicates with 10,000, 1000, or 100 HepG2 cells each and compared the ChIP-profiles around known target regions. We found that RELACS can produce robust and reproducible profiles down to 100 cells for histone marks and 1000 cells for transcription factors, beyond which the signal-to-noise ratio deteriorates (Fig. 3). To quantify signal reduction with decreasing cell numbers, we computed the fraction of reads that overlap ENCODE annotated peak regions and found that it ranges from 60% for 100,000 cells to 20% for 100 cells (Supplementary Fig. 9a). Simple peak calling with default parameters generates consistent regions between the traditional protocol with 100,000 cells and the RELACS protocol with only 1000 cells (Supplementary Fig. 9b).

### RELACS multiplexed over tissues

**Fig. 4:**
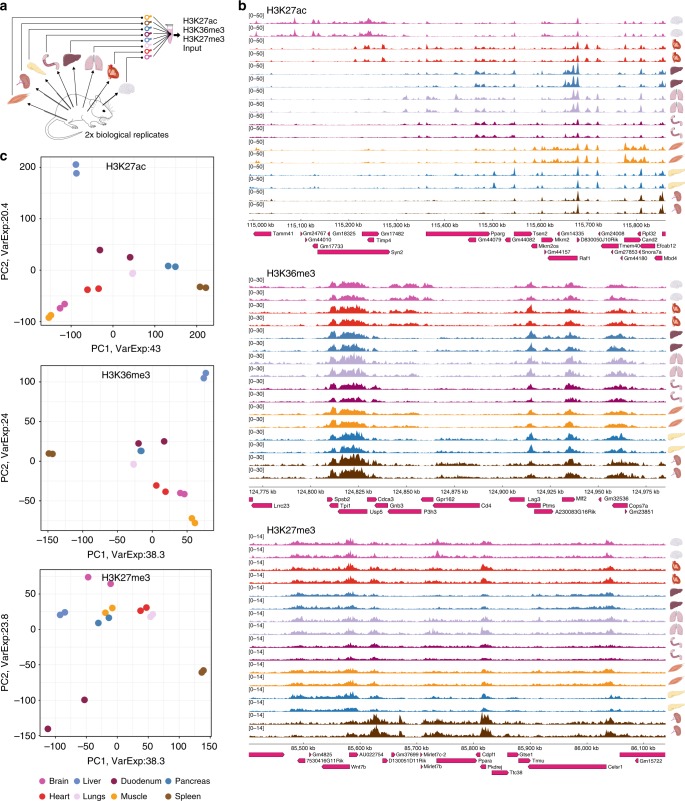
Analysis of multiple tissues and biological replicates in a single ChIP. **a** Schematic representation of the high-throughput RELACS ChIP-seq experiment. Eight mouse organs (brain, heart, lungs, liver, duodenum, pancreas, spleen, skeletal muscle) were extracted from two wild-type mice. Organs were independently homogenized, fixed, and nuclei were extracted. After chromatin digestion inside intact nuclei, different hairpin nuclear barcodes were used to mark nuclei extracted from each organ. After barcoding nuclei were pooled and lysed to release chromatin. Chromatin was split to investigate three histone modifications (H3K27ac, H3K36me3, H3K27me3) plus input for normalization control. Spleen icon is licensed under the Creative Commons Attribution 4.0 International license (Author: DataBase Center for Life Science (DBCLS), Source: http://togotv.dbcls.jp/ja/togopic.2014.20.htmL). Mouse icon is licensed under Creative Commons Attribution-Share Alike 4.0 International license (Author: Gwilz, Source: self-published work). **b** Tracks of three different histone marks (H3H27ac, H3K36me3, H3K27me3) with 8 different mouse tissues derived from two biological replicates are shown. To generate each track 25,000 cells have been used. **c** Principal component analysis illustrates a clear and consistent separation of tissues for all three histone marks where more than 60% of variation is explained (VarExp in the figure) by the first two components. Cases where only one replicate is visible indicate perfect overlap within the limits of resolution

**Fig. 5:**
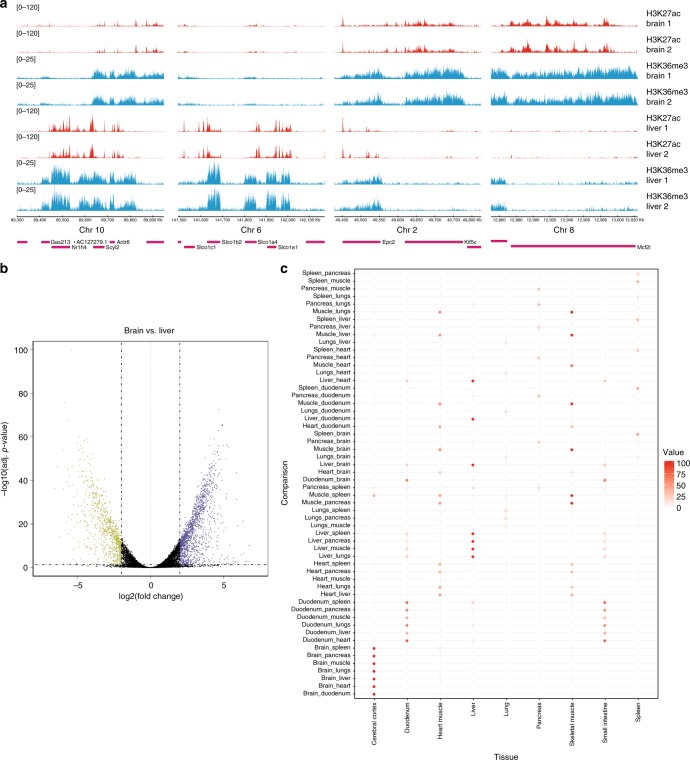
Differential analysis of histone marks in mouse tissues. **a** For 4 selected loci we show examples of differential histone marks between replicated samples from mouse brain and liver samples. Red tracks denote H3K27ac signal and green tracks are for the elongation mark H3K36me3. **b** The volcano plot illustrates the outcome of DESeq analysis of H3K36me3 read counts over annotated genes and identifies 3877 (2323) genes with increased (decreased) binding in brain vs. liver. An enrichment analysis of those gene sets reveals a highly significant enrichment in the respective tissue (*p*-value < 10^–100^). **c** Generalizing this enrichment analysis to gene sets from all pairwise comparisons (y-axis) we find the strongest enrichments always in the expected tissue. For visualization purposes, we have color-coded the enrichment score, −log_10_(*p*-value), and set a threshold of 100. Higher scores (lower *p*-values) are mapped to 100

We applied RELACS to barcode nuclei from 8 different mouse tissues and two biological replicates for a total of 16 × 25,000 cells per ChIP. For each histone mark (H3K36me3, H3K27ac, and H3K27me3) and insulator CTCF only a single ChIP on pooled-and-barcoded nuclei was performed for maximal simplicity and comparability (Fig. 4a). After sequencing, the pooled samples were demultiplexed according to the nuclear barcodes to assign all reads to their tissues of origin. For the histone marks, we observed high-quality enrichment profiles (Fig. 4b) and mapping rates, duplication rates and the FRiP scores (Supplementary Data 2). Our results are highly reproducible, as seen by clear replicate clustering in PCA plots for all analyzed histone marks (Fig. 4c). Importantly, the observed differences between pairs of tissues correspond to biological expectations. This is particularly pronounced for changes in the elongation mark, H3K36me3, over gene bodies and there is a clear enrichment for genes in the respective tissue (Fig. 5). The same analysis for CTCF was more challenging because of a much higher duplication rate (>50%) and a reduced number of usable fragments for further analysis, because at this point we cannot distinguish between technical PCR artefacts and duplicates that emerge by chance from narrow genomic regions. Nonetheless, we also see a clear signal of CTCF around known target sites (Supplementary Fig. 10). As shown in Supplementary Fig. 11, the incorporation of unique molecular identifiers (UMI) will help to retain a larger fraction of duplicates, especially in highly occupied regions such as CTCF peaks, but we leave this for future investigations.

A control sample (input) is prepared for each ChIP experiment, which not only accounts for the usual biases in the protocol, but also faithfully conveys the differences in initial sample amount and barcoding efficiency. ChIP signals of each multiplexed sample can, therefore, be normalized against their respective input controls and signals between samples can be quantitatively compared. Using multiplexed H3K36me3 ChIPs and inputs on identical HepG2 samples, we show that we can reproduce the relative scaling factor (which should be 1) within 10% (Supplementary Fig. 12a). This same procedure is then applied to multiplexed tissues, to allow proper normalization and quantitative comparisons between them (Supplementary Fig. 12b). Notice that this is an alternative to the spike-in of chromatin from other species chromatin to starting chromatin as proposed by^30^. In some cases (e.g. brain), we observe consistently high scaling factors, indicating that these tissues may have overall higher levels of H3K36me3 compared to others.

## Discussion

We have introduced an ultra-parallel and highly standardized approach to the generation and analysis of ChIP-seq data. It can be established easily in any molecular biology lab without specific specialized equipment. Compared to MNase-based protocols (e.g., Mint-ChIP^3^), RELACS is simpler, as it does not need strict control of MNase concentrations to prevent overcutting open chromatin regions and to ensure result consistency^31^. Unlike Mint-ChIP, it can also be applied to fixed samples, allowing profiling of transcription factors and co-factors easily lost by native ChIP approaches. The ability to label and pool large number of samples opens up the possibility to conduct controlled ChIP-seq experiments with very small cell numbers. We have obtained reliable and reproducible histone profiles for as few as 100 cells and transcription factor profiles from 1000 cells. We do not exclude the possibility that ChIP using even lower cell numbers can be performed, by merging a higher number of barcoded small cell populations, or by adding un-barcoded carrier chromatin prior to immunoprecipitation (to reduce the noise associated with low input samples)^10,32^.

Compared to the iChIP protocol^2^ for small cell numbers, RELACS uses intact nuclei throughout chromatin fragmentation and barcoding. This eliminates the need for an additional ChIP step just to purify chromatin prior to barcode ligation. As a result, RELACS provides a simple and highly standardized workflow for all cell types and a wide range of sample and cell numbers.

We have carefully investigated the potential limitations and biases due to the restriction site based RELACS methodology. Overall, the genome coverage at a given sequencing depth is comparable to a traditional approach, with slightly reduced coverage of the heterochromatic region and repetitive regions (due to reduced cutability and accessibility) and concomitantly increased coverage of many types of open regions. As with all other ChIP-seq protocols, we also observe a bias towards open chromatin^33^, where most of the studied marks are located (active histone marks, CTCF, p300). These biases are accounted for by using input controls, which is already done in standard algorithms for peak calling. For transcription factors (CTCF) the RELACS protocol is more sensitive, and for co-factor p300 only the RELACS method was able to obtain a signal in known target regions. Importantly, for sharp marks, we achieved the same resolution as with sonication-based methods. In our analyses with extremely small cell numbers (<1000 cells) and sharp marks (CTCF), we observed a clear reduction in library complexity, which results in high duplication rates at a given sequencing depth. In this work, we have aggressively filtered and removed all duplicate alignments, or utilized UMI to distinguish technical PCR duplicates from biological duplicates derived by restriction digestion.

Given the broad applicability of RELACS (universality across tissues, cell numbers, histone or transcription factor ChIP-seq), we propose that it can be used as a reference protocol for the ultra-parallel handling of limited clinical samples and large-scale comparative studies. Although we have applied RELACS to multiple mouse tissues, the protocol is also compatible with more stringent purification techniques (e.g., nuclear sorting), allowing the production of epigenetic profiles from any combination of samples. Moreover, the protocol could also be integrated into other assays, such as DNA methylation analysis or chromatin conformation studies. The intra-nuclei chromatin barcoding strategy paves the way for combinatorial barcoding strategies, similarly to recent developments^20–22^, used for single cell ChIP-seq analyses.

## Methods

### Cell culture

HepG2 liver hepatocellular carcinoma (ATCC, HB-8065^TM^) were cultured in Eagle’s minimal essential medium (EMEM, Lonza, 06-174) supplemented with 10% fetal bovine serum (Sigma), 2 mM L-glutamine (Lonza), 1.8 mM CaCl_2_, 1 mM sodium pyruvate (Lonza) and penicillin–streptomycin mixture (100 units/mL, Lonza), at 37 °C at 5% CO_2_ in 10 cm plates, up to 80% confluency. S2 cells were provided by Asifa Akhtar’s lab (MPI-IE) and were cultured in Express Five SFM (Thermo Fisher Scientific) supplemented with glutamax, at 27 °C.

### Mouse tissues

Experiments were performed on two 9 week-old wild-type (WT) FVB/NJ littermate male mice. Mice were euthanized using carbon dioxide (CO_2_) before organ extraction. Dissected organs have been placed in room temperature D-MEM prior to fixation. All animal studies were performed with the approval of the local authority (Regierungspräsidium Freiburg, Germany).

### Cell fixation for cell line and tissues

Adherent HepG2 and S2 cells were fixed in 1% methanol-free formaldehyde (Thermo Scientific, 28906) in D-MEM (for HepG2 cells) or Express Five SFM (for S2 cells) for 15 min at room temperature under gentle shaking. Formaldehyde was quenched for 5 min by adding 125 mM glycine final concentration. Cells were rinsed twice with ice-cold PBS, harvested by scraping and pelleted (300 g, 10 min, 4 °C). For tissues, a small portion of each organ was finely chopped. Organ fragments were transferred into a Dounce homogenizer (loose pestle), covered with 1 mL of 1% formaldehyde in D-MEM, and dissociated with 3–4 strokes, followed by 15 min incubation at room temperature. During incubation, the tissue suspension was filtered using a nylon 70 µM cell strainer (Falcon, 352350) to remove larger debris. 125 mM glycine final was added to the sample and the suspension was pelleted for 5 min at 500 g. Cell pellets were washed twice in PBS supplemented with a protease inhibitor cocktail (Roche, 11873580001) and aliquoted. Fixed cell pellets were stored at −80 °C until further usage.

### Chromatin preparation (sonication-based protocol)

Chromatin from HepG2 fixed cell pellets was prepared with the standardized method used for DEEP/IHEC consortia epigenome production, as previously described^5^. Briefly, nuclei were extracted using sonication (NEXSON) under these sonicator parameters: 75 W peak power, 2% duty factor, 200 cycles/burst, for 90 s of treatment (Covaris E220 and 1 mL tubes, cat. No. 520130). Nuclei were pelleted, followed by sonication in shearing buffer (10 mM Tris-HCl pH 8, 0.1% SDS, 1 mM EDTA) using these sonicator parameters: 140 W peak power, 5% duty factor, 200 cycles/burst, 15 min of treatment, 1 mL tubes. A chromatin aliquot was de-crosslinked, purified, and quality controlled for DNA concentration (Qubit DNA HS, Invitrogen, Q32851) and size distribution using capillary electrophoresis (Fragment Analyzer, NGS 1-6000 hs DNA kit).

### Nuclear barcodes construction

The sequence of hairpin barcode adapters is reported in Supplementary Table 2. Ultramer oligonucleotides were purchased from IDT (Integrated DNA Technology). Lyophilized oligos were resuspended in annealing buffer (10 mM Tris-HCl, pH 8, 50 mM NaCl, 1 mM EDTA) to a final 100 mM concentration. Prior to hairpin formation, oligos were diluted to 15 mM in annealing buffer. For annealing, diluted oligos were heated at 95 °C for 2 min in a thermoblock and afterward switched off and slowly cooled down to room temperature.

### RELACS barcoded chromatin preparation

RELACS involves these key steps: nuclei extraction using sonication, nuclei swelling, nuclei digestion, nuclei wash, nuclei barcoding, nuclei pooling, and chromatin release.

### Nuclei extraction by sonication (NEXSON)

Formaldehyde-fixed pellets were resuspended in 1 mL of lysis buffer (10 mM Tris-HCl pH 8, 10 mM NaCl, 0.2% Igepal) supplemented with 1× protease inhibitor cocktail (Roche, 11873580001). Nuclei were extracted by sonication using the NEXSON approach^5^. For all cell types, parameters for nuclei extraction were the following: 75 W peak power, 2% duty factor, 200 cycles/burst, 1 mL tubes (cat. No. 520130) using the Covaris E200 sonicator. Treatment time was stopped when nuclei extraction was satisfactory (over 70% of isolated nuclei). The following treatment times was used: 60–120 s (HepG2), 30 s (S2 cells), 45 s (liver, brain, spleen, lungs, skeletal muscle, duodenum), 30 s (pancreas, heart). After nuclei extraction, a small aliquot of nuclei was set aside to estimate DNA concentration and cell number from tissue samples: for this purpose, nuclei aliquots were shortly sonicated, de-crosslinked and DNA was purified using columns.

### Nuclei swelling

Approximately 500,000 nuclei were pelleted (1000 × *g*, 5 min), resuspended in 50 µL of 0.5% SDS and incubated at room temperature for 10 min. SDS was quenched adding 25 µL of 10% Triton X-100 and 145 µL of water. 25 µL of restriction enzyme buffer (CutSmart, NEB B7204S) and 2.5 µL of 100× protease inhibitor cocktail was added prior to enzyme incubation.

### Nuclei digestion

Five units of the restriction enzyme CviKI-1 (NEB, R0710S) were added to every 100,000 nuclei. Samples were digested for 16 h at 20 °C in a thermomixer under shaking (800 rpm). Note that the restriction endonucleases (used in RELACS) lack exonuclease activity and are easier to titrate upon variation of the input material. The same amount of enzyme (20 U) can be used to digest approximately 10,000 to one million fixed cells without affecting chromatin quality and ChIP.

### Nuclei wash

Restriction enzymes were removed by pelleting the nuclei (1000 × *g*, 5 min) and then washing in 200 µL of nuclei wash solution (10 mM Tris-HCl, pH 8, 0.25% Triton X-100, 0.1 mg/mL BSA). At this stage 10–20 µL of nuclei were set aside for digestion quality control. Remaining nuclei were pelleted down and resuspended in 10 mM Tris-HCl, pH 8 (25 µL per 100–100,000 nuclei) for barcoding.

### Nuclei barcoding

Digested chromatin inside nuclei was end-repaired and A-tailed using end-repair and A-tailing components from NEBNext Ultra II DNA library preparation kit (E7645L, NEB) with the following modifications, to enhance efficiency and to reduce handling volumes: 1.5 µL of end prep enzyme mix and 3.5 µL of reaction buffer were added to each 25 µL digested nuclei aliquot. Samples were mixed and incubated at 20 °C for 30 min followed by heat inactivation at 65 °C for 5 min. 1.2 µL of hairpin adapter nuclear barcodes were added to each sample. Ligation of barcodes to A-tailed chromatin was performed using components from NEBNext Ultra II DNA library preparation kit, with the following modifications: per sample 15 µL of ligation master mix and 0.5 µL of ligation enhancer were added, followed by 15 min incubation at 30 °C and 15 min incubation at 20 °C. Barcoding efficiency is consistent between 100 and 500,000 nuclei using an unmodified protocol.

### Nuclei pooling and chromatin release

Ligase was inhibited by adding 4.7 µL of 3 M NaCl into each well. All barcoded nuclei were pooled and pelleted down (5000 × *g*, 10 min, 20 °C). The supernatant was removed and re-centrifuged at a higher speed to increase recovery (11000 × *g*, 5 min, 20 °C). Nuclei pellets were combined and resuspended in shearing buffer (10 mM Tris-HCl pH 8, 0.1% SDS, 1 mM EDTA; approx. 130 µL per up to 500,000 nuclei maximum). Chromatin was released by sonication-assisted nuclei lysis by treating the samples for 5 min using these parameters: peak power 105 W, 2% duty factor, 200 cycles/burst, Covaris microtubes (520052), Covaris E220 sonicator. This barcoded chromatin was subsequently used for ChIP.

### Digestion quality control

ChIP elution buffer (10 mM Tris-HCl, pH 8, 1 mM EDTA, 1% SDS) was added to each nuclei aliquot to a final 100 µL volume. Samples were de-crosslinked and deproteinized by adding 4 µL of 5 M NaCl, 2 µL 10 mg/mL DNase-free RNase A and 2 µL of 20 mg/mL proteinase K and incubating 30 min at 37 °C followed by 65 °C incubation for a minimum of 2 h. Samples were purified using Qiagen PCR purification kit columns. DNA was quantified using a Qubit DNA HS kit and fragment size distribution monitored by capillary electrophoresis. Cell number for tissue samples was estimated from the total DNA amount, taking into account that each mouse diploid cell contains approximately 6.6 pg of DNA.

### Antibodies

The following antibodies targeting histone modifications were used for ChIP-seq (1 μg per ChIP for cell numbers below 100,000, 2 μg per ChIP for cell total cell numbers per ChIP below 250,000): anti-H3K27ac (C15410196, lot A1723–041D), anti-H3K4me3 (C15410003, lot A5051–001P), anti-H3K4me1 (C15410194, lot A1863–001D), anti-H3K36me3 (C15410192, lot A1847–001P), anti-H3K9me3 (C15410193, A1671–001P), anti-H3K27me3 (C15410195, lot A1811–001P), all from Diagenode. Transcription factors antibodies: CTCF (3 μg/ChIP, Abcam, ab70303), p300 (10 μg/ChIP, Santa Cruz, sc-585).

### ChIP

RELACS or sonication-based chromatins were diluted 1:2 in 1× buffer iC1 from iDeal ChIP-seq kit for histones (Diagenode, C01010173) and supplemented with 1× protease inhibitor cocktail (Roche complete EDTA-free, 11873580001). Diluted chromatin was divided into 200 µL aliquots and the antibody of interest has been added. For transcription factor ChIP, an additional 2.6 µL of 5 M NaCl was added to each ChIP to adjust the final salt concentration to 140 mM. Automatic ChIP was performed using the SX-8G Compact IP-Star platform (Diagenode), immunoprecipitation buffers from the iDeal ChIP-seq kit for histones and A-conjugated magnetic beads (Diagenode, C03010020). The following IP-Star pre-programmed parameters were used for automated ChIP: indirect ChIP method, 200 µL ChIP volume. Immunoprecipitation was carried out by incubating chromatin with the antibody for 10 h at 4 °C. Immunocomplexes were captured by protein-A conjugated magnetic beads (3 h of beads incubation at 4 °C) and washed four times using iDeal 1–4 wash buffers (5 min of incubation at each wash). After ChIP, eluates were recovered manually, RNase A-treated, de-crosslinked and deproteinized for 30 min at 37 °C and 4 h at 65 °C. DNA was purified using Qiagen MinElute columns (Qiagen, 28006) with a final elution in 20 µL.

### Library amplification for RELACS ChIP-seq samples

18 µL of RELACS ChIP DNA was mixed together with 25 µL of PCR master mix from NEBNext Ultra II DNA library preparation (E7645L, NEB), 3 µL of USER enzyme and 2 µL of 10 μM universal PCR primer. 2 µL of 10 μM indexed primers were added to each respective ChIP. Hairpin adapters were opened by a 15 min incubation at 37 °C. 12 PCR cycles were used for enrichment of ligated fragments using the following program: hot start 98 °C 30 sec. Amplification: 98 °C 10 sec, 65 °C 75 sec. and a final extension at 65 °C for 5 min. PCR-amplified samples were purified twice using Ampure XP beads (Beckman Coulter) at 0.9 and 1× ratio.

### Traditional ChIP-seq library preparation

For each library, 1–5 ng of purified ChIP DNA was used. Libraries were prepared using the NEBNext Ultra II DNA Library Prep kit for Illumina (E7645L, NEB) with the following modifications. After adapter ligation, size selection was omitted and adapter-ligated fragments were purified using AMPure XP beads (Beckman Coulter) at 1× ratio. USER enzyme treatment was performed together with PCR enrichment using the following program: 15 min incubation at 37 °C, hot start 98 °C for 30 sec., then 12 cycles of 98 °C for 10 sec and 65 °C for 75 sec. A final extension was done at 65 °C for 5 min. Amplified libraries were purified twice using 0.9 and then 1× Ampure XP beads.

### Library quality control and sequencing

All libraries were quality-controlled for DNA concentration (Qubit DNA HS, Invitrogen, Q32851) and size distribution (capillary electrophoresis Fragment Analyzer, NGS 1-6000 bp hs DNA kit). Adapter-cleaned libraries were normalized to the desired molarity and then pooled accordingly with a 10% PhiX spike-in. Library pools were denatured and prepared for instrument loading following Illumina guidelines. Samples were sequenced paired-end with a read length of 75 bp on an Illumina HiSeq 3000 instrument.

### Demultiplexing, mapping, and quality control

Using an in-house written script the sequences were demultiplexed. Sequences without barcode or having more than one mismatch between barcodes on each paired-end mate were discarded from further analysis. HepG2 reads were mapped to the hs37d5 reference human genome and mouse reads were mapped to mm10 reference genome. snakePipes DNA-mapping workflow v0.3.2.1 (https://zenodo.org/badge/latestdoi/54579435) was used to compute quality controls using FastQC (https://www.bioinformatics.babraham.ac.uk/projects/fastqc/), trim the reads using TrimGalore! (https://www.bioinformatics.babraham.ac.uk/projects/trim_galore/) and align the reads using bowtie2–2.2.8^34^ with the following configurations: -X 1000 --local --fr --rg. Next, duplicate reads and insert size distributions were determined using picard-1.136. Additional quality metrics were generated using estimateReadsFiltering from deepTools^35^ v3.0. MultiQC/1.3^36^ was then used to selectively combine the outputs from the above steps (Supplementary Data 1–4).

### Filtering and normalization

Coverage and normalization to the input was obtained using deepTools-2.5.4, bamCoverage (--normalizeTo1x 2451960000, --extendReads, --ignoreDuplicates, --blackListFileName) and bamCompare (--missingDataAsZero, --skipZeros, --blackListFileName) respectively. Blacklists for mouse and human were obtained from the ENCODE project^37^. The human blacklist (http://hgdownload.cse.ucsc.edu/goldenPath/hg19/encodeDCC/wgEncodeMapability/) was supplemented with unplaced contigs and decoy sequences from the 1000 genome projects. FRiPs scores were obtained using deepTools/plotEnrichment^35^. In all downstream analyses, duplicates and blacklisted regions were removed, and all results were computed using fragments (parameter --extendReads).

### Visualization

Genome track plots were generated using the normalized bigWig files returned from bamCompare using deepTools/pyGenomeTracks (http://github.com/deeptools/pyGenomeTracks). All heatmaps and profiles were generated using deepTools. Sharp marks (CTCF, p300, H3K4me3, H3K27ac) were aligned around the center of the peak (computeMatrix reference-point), while other marks were considered broad and scaled to the region (computeMatrix scale-regions). In each case, flanking regions were added to show the signal away from enriched regions. computeMatrix was executed with the following parameters: --missingDataAsZero, --skipZeros, and --blackListFileName, while flanking regions (-a,-b) were defined according to the nature of the mark (broad 50 kb, narrow 3 kb).

### PCA plots

Principal component analysis was performed on the log2 ratio of the ChIP to the input (bigWig). First, for each sample a genome-wide coverage was computed in windows of 10 kb (deepTools/multiBigwigSummary bins, bs = 10000) and blacklisted regions were filtered. In R/3.4.1, prcomp was used to compute the PCA over the top 5000 most variable bins.

### Identification of restriction site position

Restriction sites were identified using the Bio.Restriction package from Biopython on the human assembly GRCh37 and mouse assembly GRCm38. In total, 44,340,181 sites and 39,483,329 sites were identified, respectively.

### Computation of restriction site bias

To identify if restriction site frequencies varied at different genomic regions, we computed the overlap between the total set of restriction sites and the different chromatin states for HepG2^38^ using coverageBed from bedtools2/2.27.0^39^. The number of restriction sites per kbp was then computed using the sum of all observed overlaps divided by the sum of the respective peak length in kbp.

### Computation of RPKM for ChIPs

Read enrichment in HepG2 was assessed via FRiP scores. First, using deeptools/plotEnrichment (--extenReads --ignoreDuplicates), the fraction of reads, that fall into peaks was computed and obtained as a percentage. The FRiP scores were divided by the fraction of the genome falling into peaks and then multiplied by 1 M to obtain RPKM values that are comparable across regions.

### Peak calling

Sharp peaks were identified using MACS2/2.1.0^27^, with the following parameters: -f BAM, --nomodel, -g 2900338458. The parameter --extsize was given as the median fragment length computed using Picard. For broad marks, enriched regions were called using histoneHMM/histoneHMM_call_regions.R^28^ with bin size = 750 and -P 0.1.

### Peak compilations

Human peak annotations were obtained from ENCODE projects (GSM1003519, GSM733685, GSM733737, GSM733743, GSM733754, GSM798321, GSM803499, GSM822287, GSM935545). Peaks in blacklisted regions were removed and overlapping regions were merged. For broad marks (H3K4me1, H3K36me3, H3K27me3, H3K9me3), regions shorted than 1000 bp were discarded as the frequently denote intermittent enrichments in much larger domains. Peak annotations for 18 different mouse cell types were obtained from the group of Bing Ren^40^: http://chromosome.sdsc.edu/mouse/download.htmL. We used the liftOver tool to convert this data from mm9 to mm10 and merged peaks of the same type (e.g. CTCF) if they were within 20 bp of each other. Finally, we only used merged peaks that were present in at least 2 cell types.

### Motif analysis

We annotated the human genome (hs37d5) with the CTCF motif matrix (MA0139.1) defined by the JASPAR database^41^. This motif was converted into a position-specific score matrix and a position-specific energy matrix, as described in^42^. The TRAP program was used to predict the binding affinity of CTCF for bins of 50 bp quantitatively. The same software was used to predict also binary motif instances using a pre-calculated (balanced) score threshold of 6.05. The latter was used to calculate the fraction of CTCF peaks harboring a motif, while the logarithm of the predicted affinity is used for visualization of binding strength in heatmaps (Fig. 2d).

### Differential analysis of histone marks

For the analysis of differential histone maps in mouse tissues we used a list of all protein-coding genes from Ensembl GRCm38 (release 91) and counted the reads of histone marks over those gene bodies, using bedtools multicov^39^. The corresponding count matrix was used as input for DESeq2 version 1.20.0^43^. At the same time, we have used Input-control data to generate another count matrix from which we estimate the appropriate size factors to be used for further DESeq analysis of histone marks. Analysing all pairwise contrasts among the 8 selected tissues with strict thresholds (q < 0.05 and abs(log2-fold change)>2), we identified sets of differentially modified genes. Those sets were subjected to a systematic analysis of tissue enrichment, using TissueEnrich^44^. We converted the p-values reported by TissueEnrich to an enrichment score, −log_10_(*p*-value), and defined threshold of 100, beyond which we set all scores to 100.

### Chromatin states

Chromatin states were obtained from the UCSC table browser^45^. The 15 chromatin states were obtained using chromHMM for the HepG2 cell line^38^. Chromatin state 15 was not used, as it mostly contained human blacklisted regions.

### Code availability

The basic mapping and QC were done using v0.5 of snakePipes^46^ for DNA-mapping and ChIP-seq, with only a minor modification to adjust for local mapping in the Bowtie2 step (*--local*). The workflows are freely available at https://github.com/maxplanck-ie/snakemake_workflows. The RELACS demultiplexing step was done via demultiplex_relacs.py/version 1.0, which is available at https://github.com/dpryan79/Misc/tree/master/InternalBarcodes. For customized downstream analysis we used deepTools^35^ v 2.5.4, as described above.

## Electronic supplementary material

Supplementary Information

Description of Additional Supplementary Files

Supplementary Data 1

Supplementary Data 2

Supplementary Data 3

Supplementary Data 4

## Data Availability

Data has been uploaded to the Gene Expression Omnibus (GEO) with accession code: GSE111000.
