# Supplementary material for: RELACS nuclei barcoding enables high-throughput ChIP-seq
Source: Commun Biol. 2018 Dec 5;1:214. doi: 10.1038/s42003-018-0219-z (PMC6281648; doi:10.1038/s42003-018-0219-z)
Supplement: Supplementary file 1 — Supplementary Information [file 42003_2018_219_MOESM1_ESM.pdf]

Supplementary material

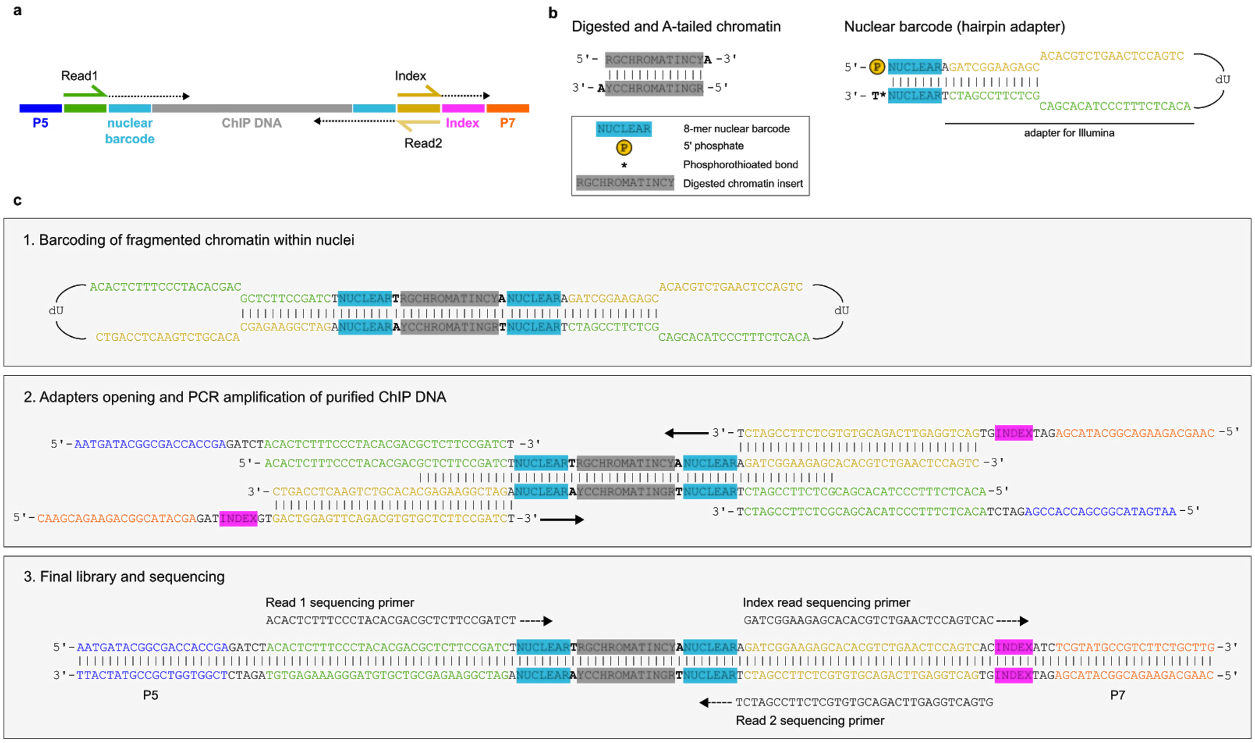

Supplementary Figure 1. (a) Schematic representation of RELACS library structure. Final libraries are identified by dual barcodes: the nuclear barcode, to identify the initial cell population, and the Illumina barcode (index), to identify the respective ChIP or input sample. Sequencing reads are indicated (dotted arrows). Both read 1 and read 2 start with the nuclear barcode read. Index read identify the Illumina barcode. **(b)** Structure of digested chromatin and hairpin adapters harboring nuclear barcodes, prior the ligation step. At both the ends of the digested chromatin the restriction site CviKI-1 is present (RG|CY, blunt cut). After adenylation one 3'-protruding A is present at both the ends of the chromatin. Hairpin adapters contain a double-strand 8-mer barcode at the end of the stem and one protruding T base at the 3' end of the hairpin, connected via phosphorothioated bond to prevent base loss. The area of the hairpin that serves as conventional adapter is highlighted (named adapter for Illumina). **(c)** Molecular details of RELACS library construction and sequencing. In step 1 is indicated the final construct of barcoded chromatin. After A-tailing of the digested chromatin, hairpin adapters are ligated to both the ends of chromatin fragments. This step occurs inside intact nuclei. Hairpin loops are left closed to prevent degradation that may occur throughout the protocol. In step 2 is indicated the PCR amplification

that occurs after ChIP and DNA purification. Using standard DNA library preparation workflow, hairpin adapters are opened at the loop site by excision of the uracil base. Primers are used to amplify and to complete the library construct. At the end of preparation final libraries (step 3) also present Illumina index and sequences necessary for illumina flow-cell clustering. Sequences of conventional oligos for Illumina sequencing are indicated.

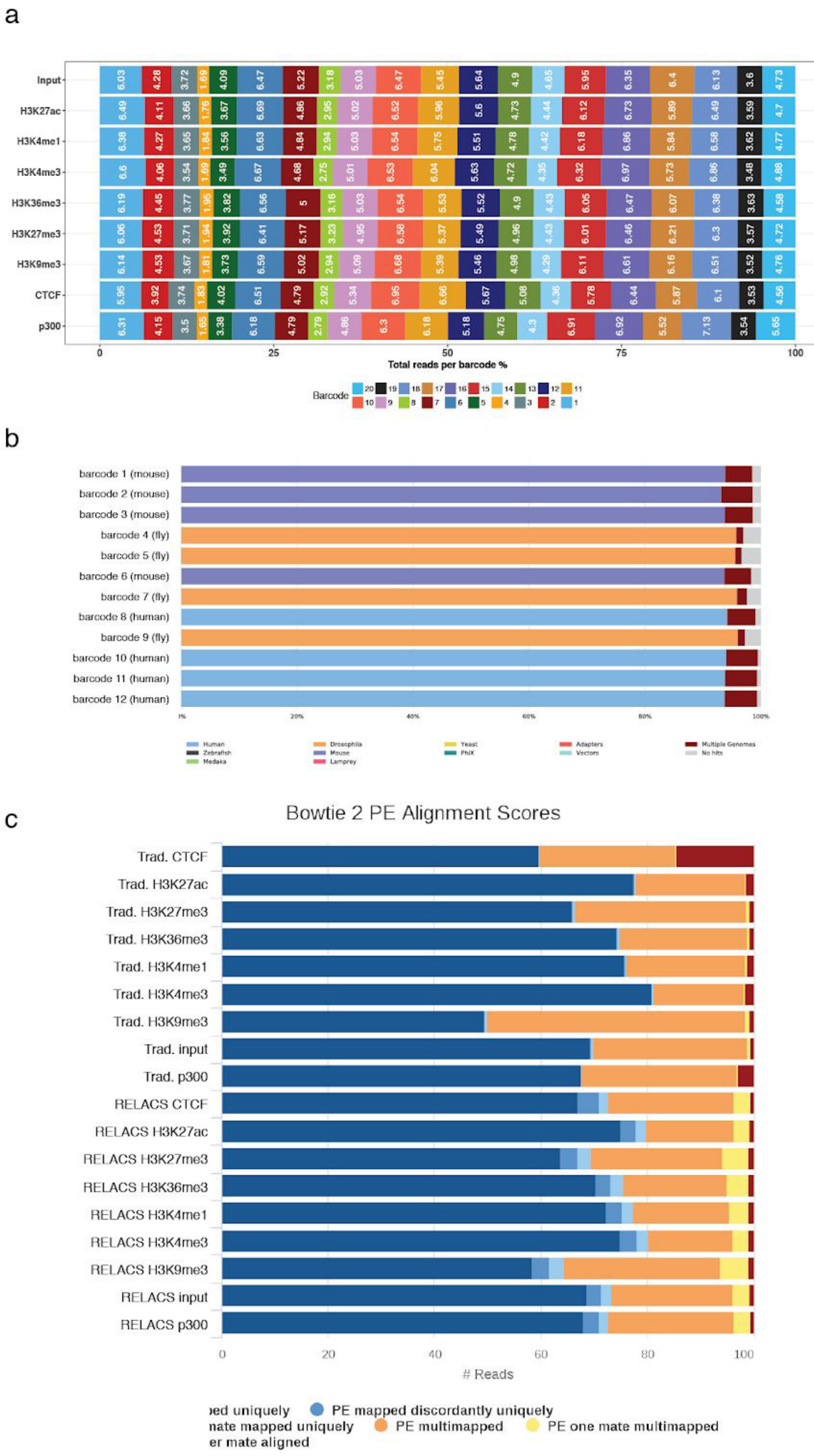

a
Input
H3K27ac
H3K4me1
H3K4me3
H3K36me3
H3K27me3
H3K9me3
CTCF
p300
0
25
50
75
100
Total reads per barcode %
Barcode
b
barcode 1 (mouse)
barcode 2 (mouse)
barcode 3 (mouse)
barcode 4 (fly)
barcode 5 (fly)
barcode 6 (mouse)
barcode 7 (fly)
barcode 8 (human)
barcode 9 (fly)
barcode 10 (human)
barcode 11 (human)
barcode 12 (human)
c
Bowtie 2 PE Alignment Scores
Trad. CTCF
Trad. H3K27ac
Trad. H3K27me3
Trad. H3K36me3
Trad. H3K4me1
Trad. H3K4me3
Trad. H3K9me3
Trad. input
Trad. p300
RELACS CTCF
RELACS H3K27ac
RELACS H3K27me3
RELACS H3K36me3
RELACS H3K4me1
RELACS H3K4me3
RELACS H3K9me3
RELACS input
RELACS p300
0
20
40
60
80
100
Reads
PE mapped discordantly uniquely
PE multimapped
PE one mate multimapped

Supplementary Figure 2. Quality controls (a) Percentage of reads belonging to each of the 20 barcodes used in HepG2 samples after demultiplexing. The relative fractions of barcodes observed in the Input sample are recapitulated in the various ChIP. **(b)** Mouse, fly and human samples (four replicates each) were labeled with different nuclear barcodes using RELACS protocol, and pooled to check for unspecific barcode re-ligation that might occur after nuclei pooling. DNA has been purified and PCR amplified to complete library construction. After sequencing samples have been de-multiplexed by the nuclear barcodes. We used fastq_screen/0.5.1 (http://www.bioinformatics.babraham.ac.uk/projects/fastq_screen/) to check for cross-contamination between samples. The absence of off-species sequences in each respective sample highlight that unspecific barcode re-ligation does not occur after the nuclei pooling step. **c.** Bowtie2 alignment results for traditional ChIP and RELACS protocols. For the RELACS samples, the 20 barcodes in HepG2 were merged. Individual alignment results can be found in Supplementary data 1. For the RELACS data set, there is an slight increase (3-5%) of disconcordantly and multi-mapping reads

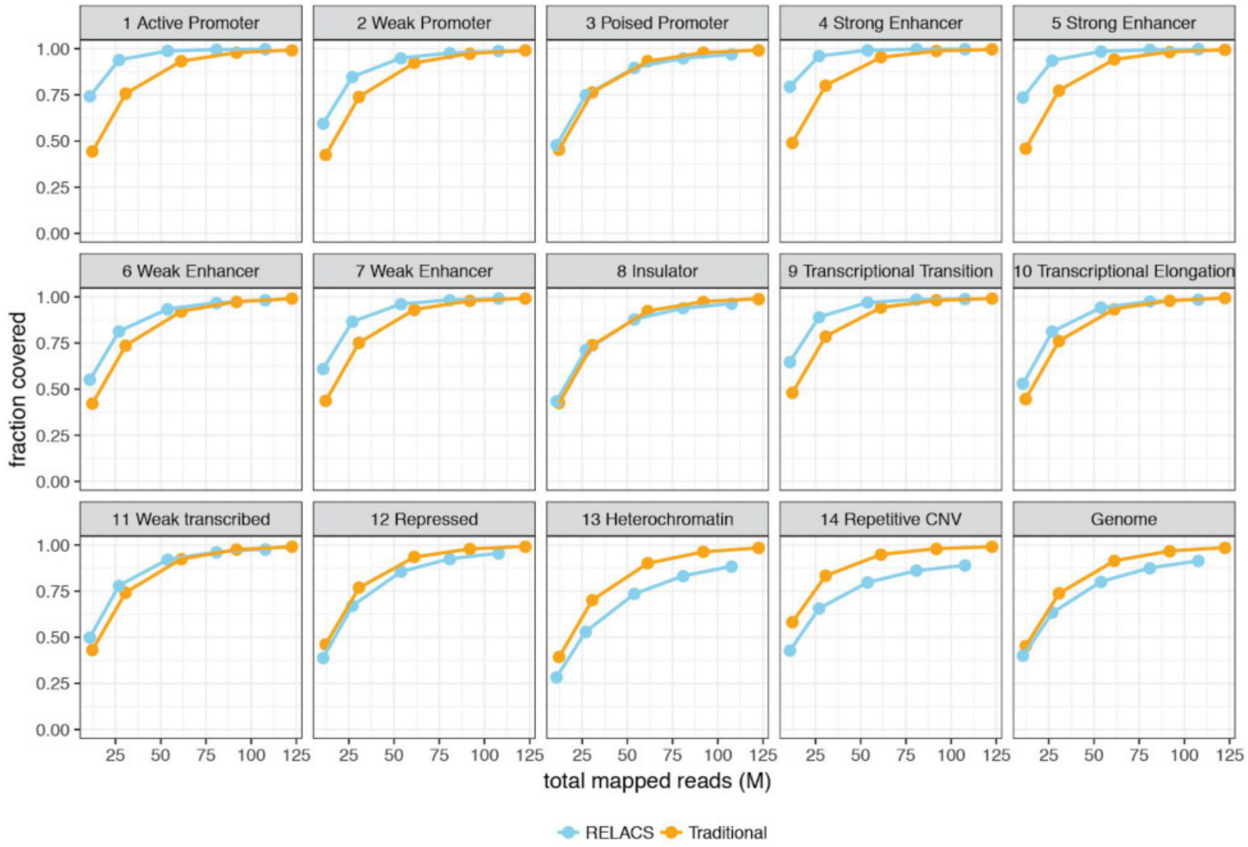

Supplementary Figure 3. Genome and region coverage vs. sequencing depth. Chromatin states based on chromHMM for HepG2 cells (see methods) were used to identify coverage with respect to sequencing depth for the RELACS and traditional protocols. The global genome coverage is shown in the last panel. The results are based on the input samples for RELACS and traditional which were subsampled using samtools. For the computation of coverage we used deepTools bamCoverage with the --extendReads option. Regions containing unspecified nucleotides (NNN) in the genome assembly were excluded.

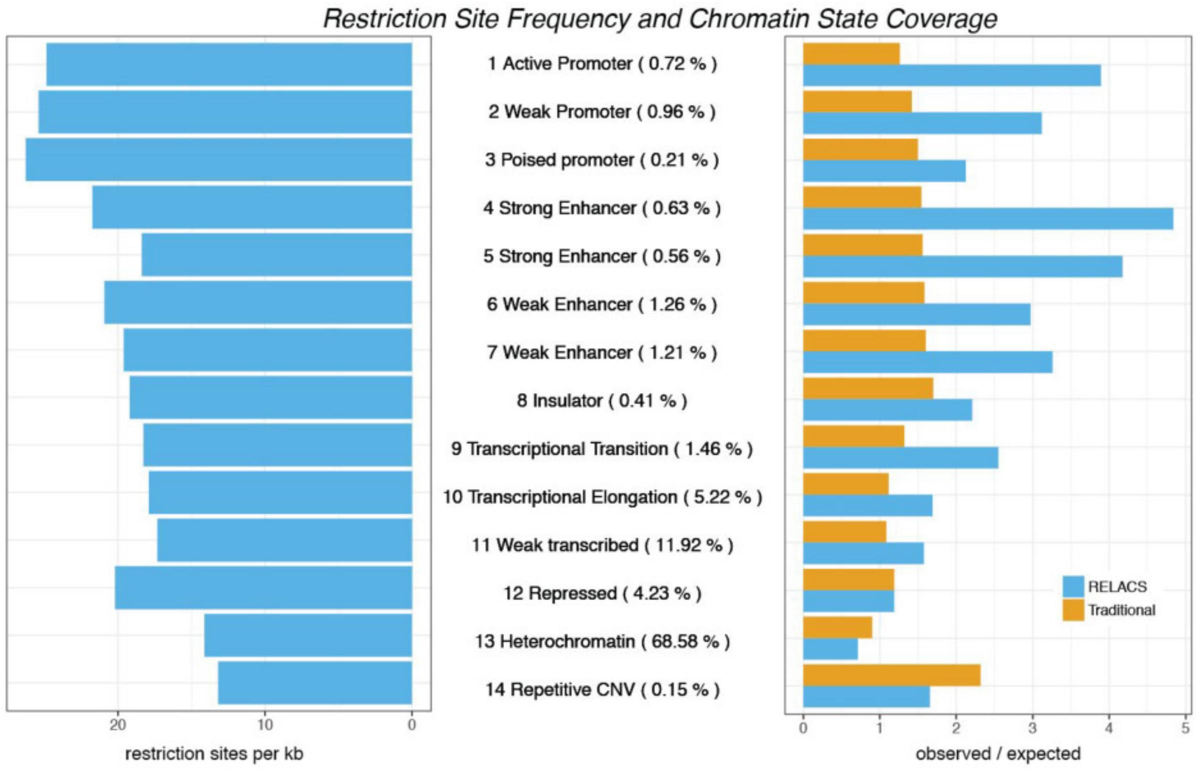

Supplementary Figure 4. Chromatin states based on chromHMM for HepG2 cells (see methods) were used to identify bias with respect to frequency of restriction sites (left) and bias with respect to accessibility of the restriction enzyme (right). In the right panel, the observed value is the fraction of reads overlapping a chromatin state and the expected value is the fraction occupied by the chromatin states in the the genome (shown as percentage in the chromatin state labels). The RELACS and traditional input samples were used to compute observed fractions.

a

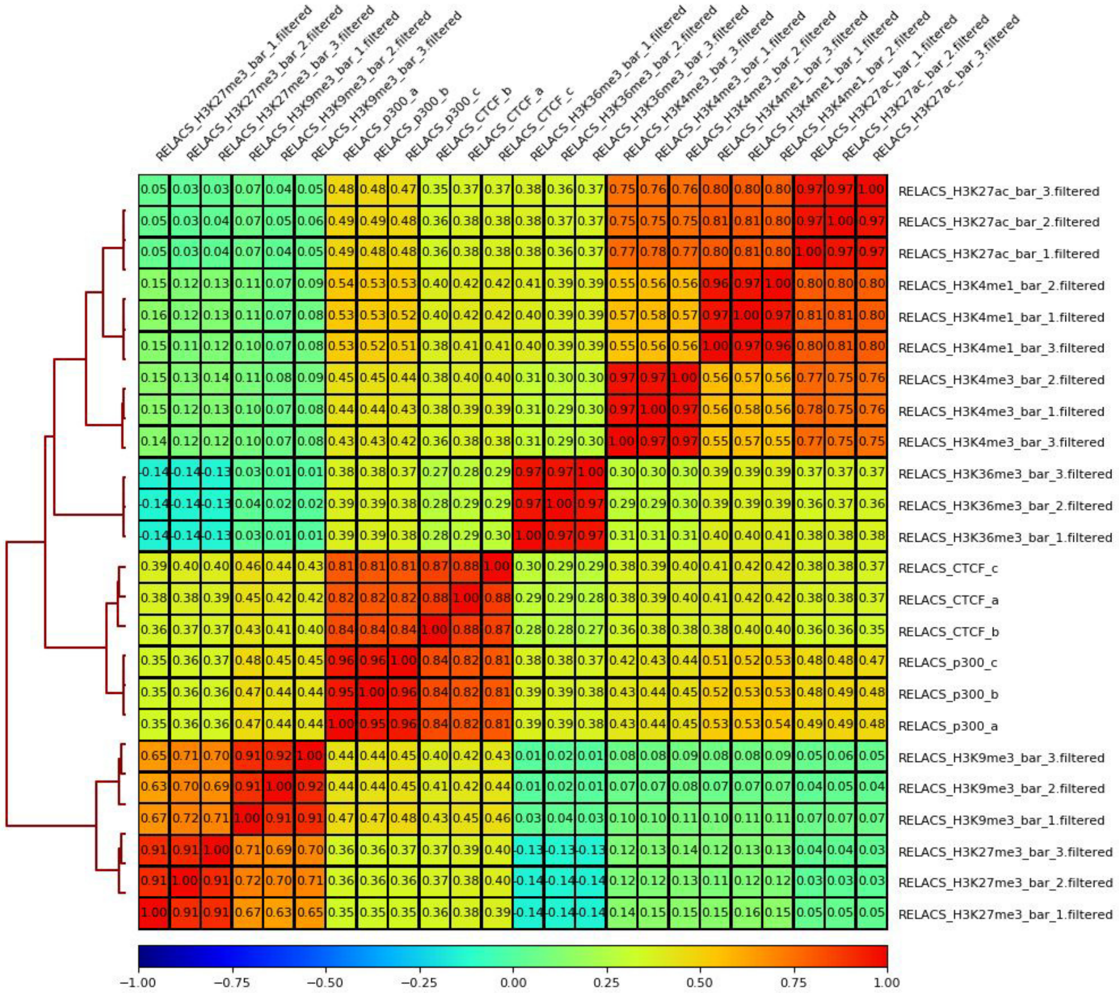
RELACS_H3K27me3_bar_1.filtered
RELACS_H3K27me3_bar_2.filtered
RELACS_H3K27me3_bar_3.filtered
RELACS_H3K9me3_bar_1.filtered
RELACS_H3K9me3_bar_2.filtered
RELACS_H3K9me3_bar_3.filtered
RELACS_p300_a
RELACS_p300_b
RELACS_p300_c
RELACS_CTCF_b
RELACS_CTCF_a
RELACS_CTCF_c
RELACS_H3K36me3_bar_1.filtered
RELACS_H3K36me3_bar_2.filtered
RELACS_H3K36me3_bar_3.filtered
RELACS_H3K4me3_bar_3.filtered
RELACS_H3K4me3_bar_1.filtered
RELACS_H3K4me3_bar_2.filtered
RELACS_H3K4me1_bar_3.filtered
RELACS_H3K4me1_bar_1.filtered
RELACS_H3K4me1_bar_2.filtered
RELACS_H3K27ac_bar_1.filtered
RELACS_H3K27ac_bar_2.filtered
RELACS_H3K27ac_bar_3.filtered
RELACS_H3K27ac_bar_3.filtered
RELACS_H3K27ac_bar_2.filtered
RELACS_H3K27ac_bar_1.filtered
RELACS_H3K4me1_bar_2.filtered
RELACS_H3K4me1_bar_1.filtered
RELACS_H3K4me1_bar_3.filtered
RELACS_H3K4me3_bar_2.filtered
RELACS_H3K4me3_bar_1.filtered
RELACS_H3K4me3_bar_3.filtered
RELACS_H3K36me3_bar_3.filtered
RELACS_H3K36me3_bar_2.filtered
RELACS_H3K36me3_bar_1.filtered
RELACS_CTCF_c
RELACS_CTCF_a
RELACS_CTCF_b
RELACS_p300_c
RELACS_p300_b
RELACS_p300_a
RELACS_H3K9me3_bar_3.filtered
RELACS_H3K9me3_bar_2.filtered
RELACS_H3K9me3_bar_1.filtered
RELACS_H3K27me3_bar_3.filtered
RELACS_H3K27me3_bar_2.filtered
RELACS_H3K27me3_bar_1.filtered
−1.00
−0.75
−0.50
−0.25
0.00
0.25
0.50
0.75
1.00

b

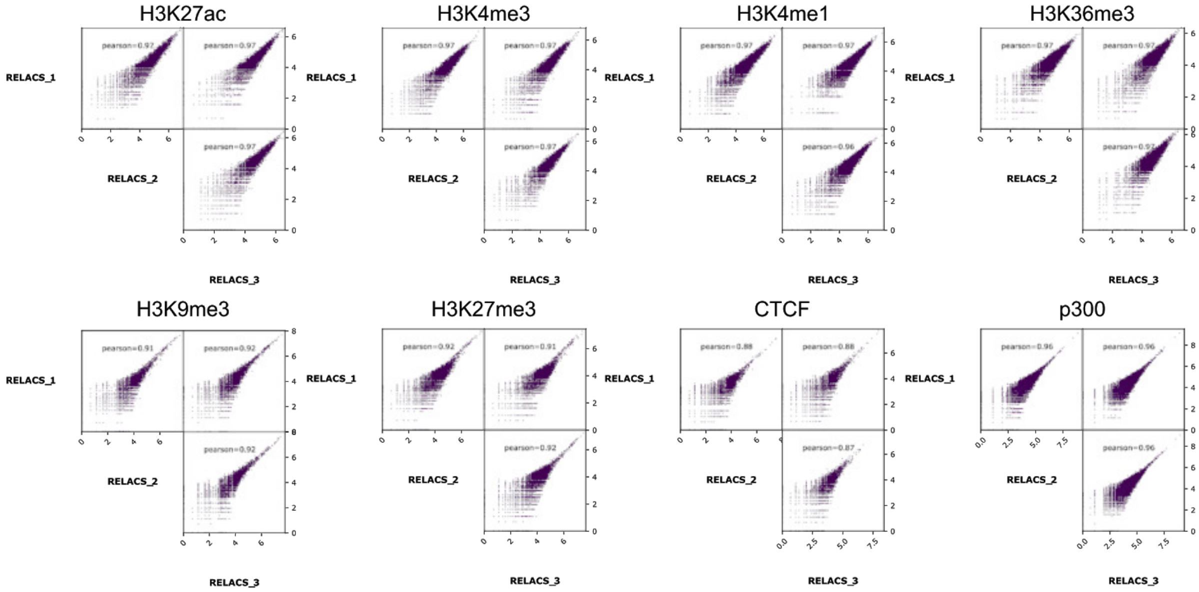

c.

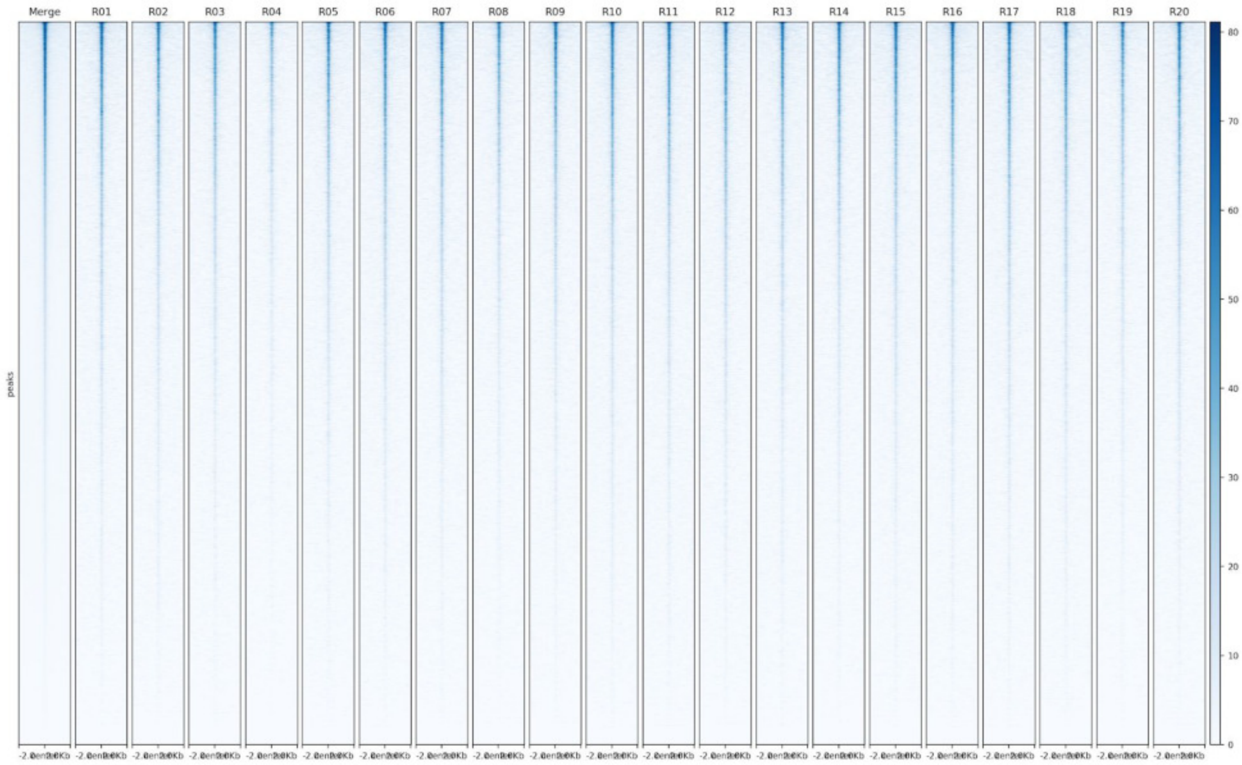

Supplementary Figure 5: Technical reproducibility.

(a) Here we show the genome-wide correlations among different samples and technical replicates from 3 representative barcodes used for HepG2 (5000 cells each). The choice of 3 (out of 20) barcodes was for practical visualization purposes. This analysis was done for bin size of 10kb. For CTCF and p300, 3 x 6

barcodes were merged, because individually they were sequenced rather shallow (<1M fragments per barcode). **(b)** For replicates corresponding to the same mark we also show genome-wide scatterplots to illustrate high technical reproducibility summarized by correlation coefficients above. **(c)** For CTCF we show the high technical concordance within ±2kb around known target regions (ENCODE peaks) for this sharp mark.

a

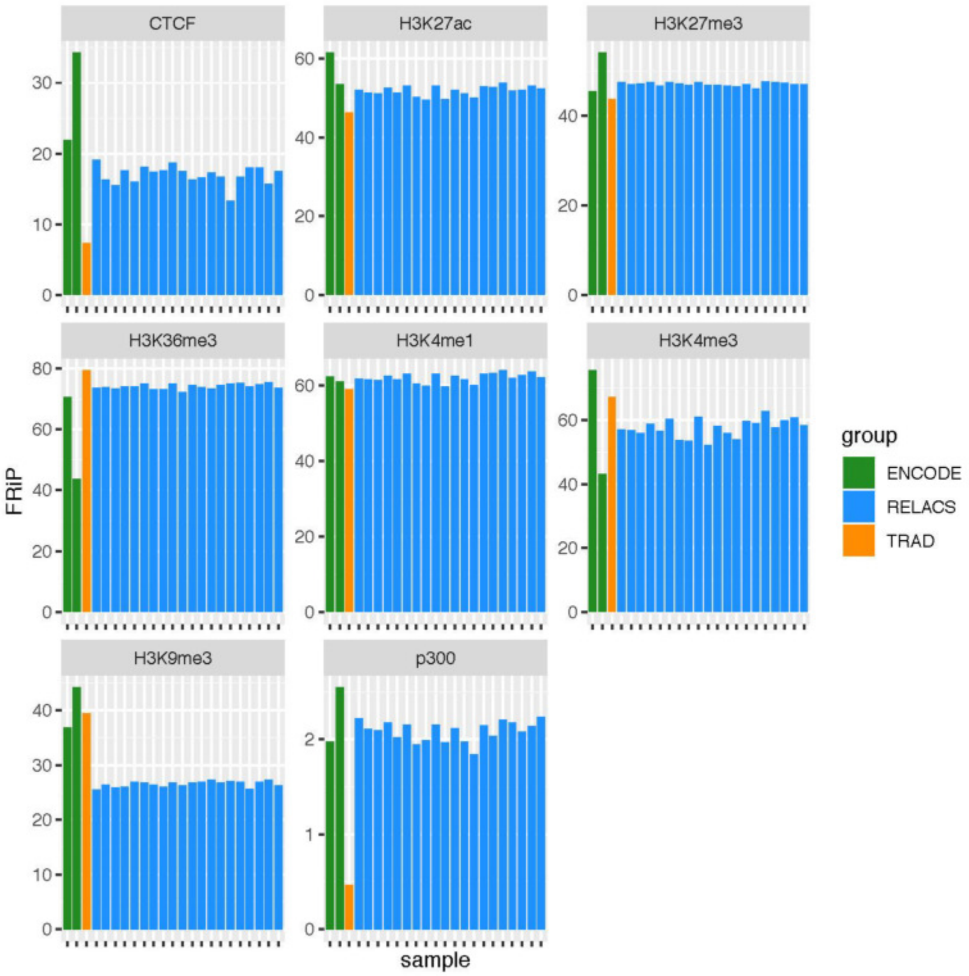
CTCF
H3K27ac
H3K27me3
H3K36me3
H3K4me1
H3K4me3
H3K9me3
p300
FRiP
sample
group
ENCODE
RELACS
TRAD

b

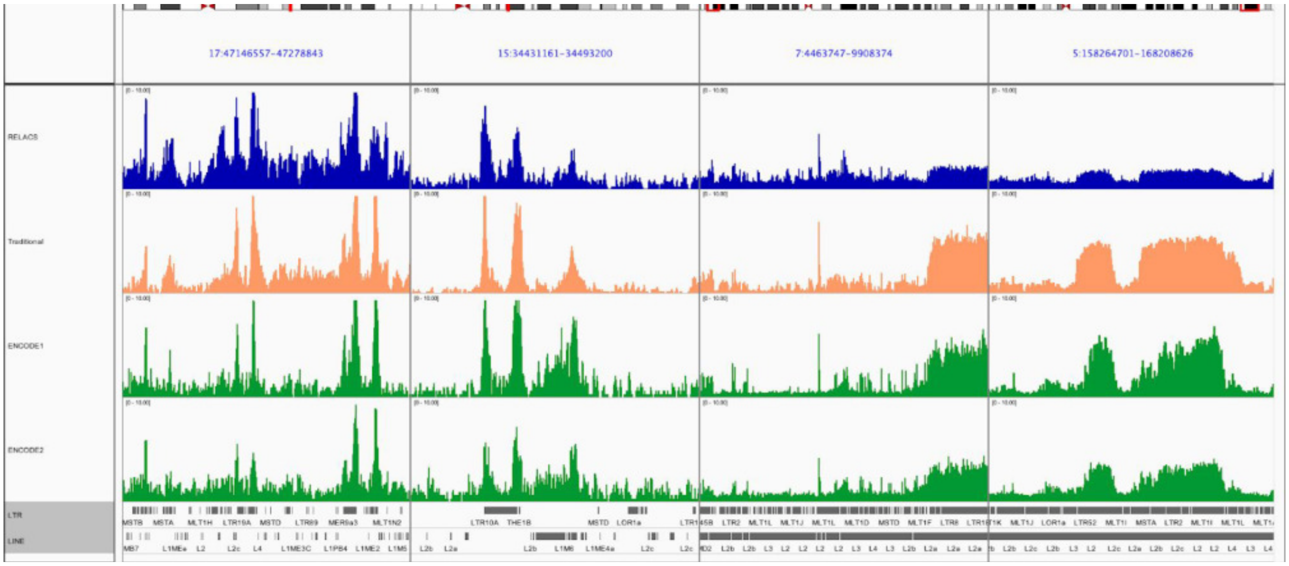
17:47146557-47278843
15:34431161-34493200
7:4463747-9908374
5:158264701-168208626
RELACS
Traditional
ENCODE1
ENCODE2
LTR
LINE

Supplementary Figure 6: FRiP scores and examples for repressed loci.

(a) The fraction of reads in peaks (FRiP in %) was calculated for our own data (1x TRADitional and 20x RELACS), and for two replicates from ENCODE data that were generated with different protocols, cell numbers and antibodies. Since the latter was used to define peaks end enriched regions, it is expected to be higher. The good overall agreement for all marks signifies a comparable signal-to-noise ratio of RELACS for most marks and much improved performance over the traditional method for more challenging marks (CTCF, p300) and smaller cell numbers (20x 5000 cells). **(b)** Selected loci for repressive H3K9me3 mark. Although reduced in strength, RELACS data for H3K9me3 captures the characteristic enrichments over repetitive elements (shown in grey are LTR and LINE) and large heterochromatic domains.

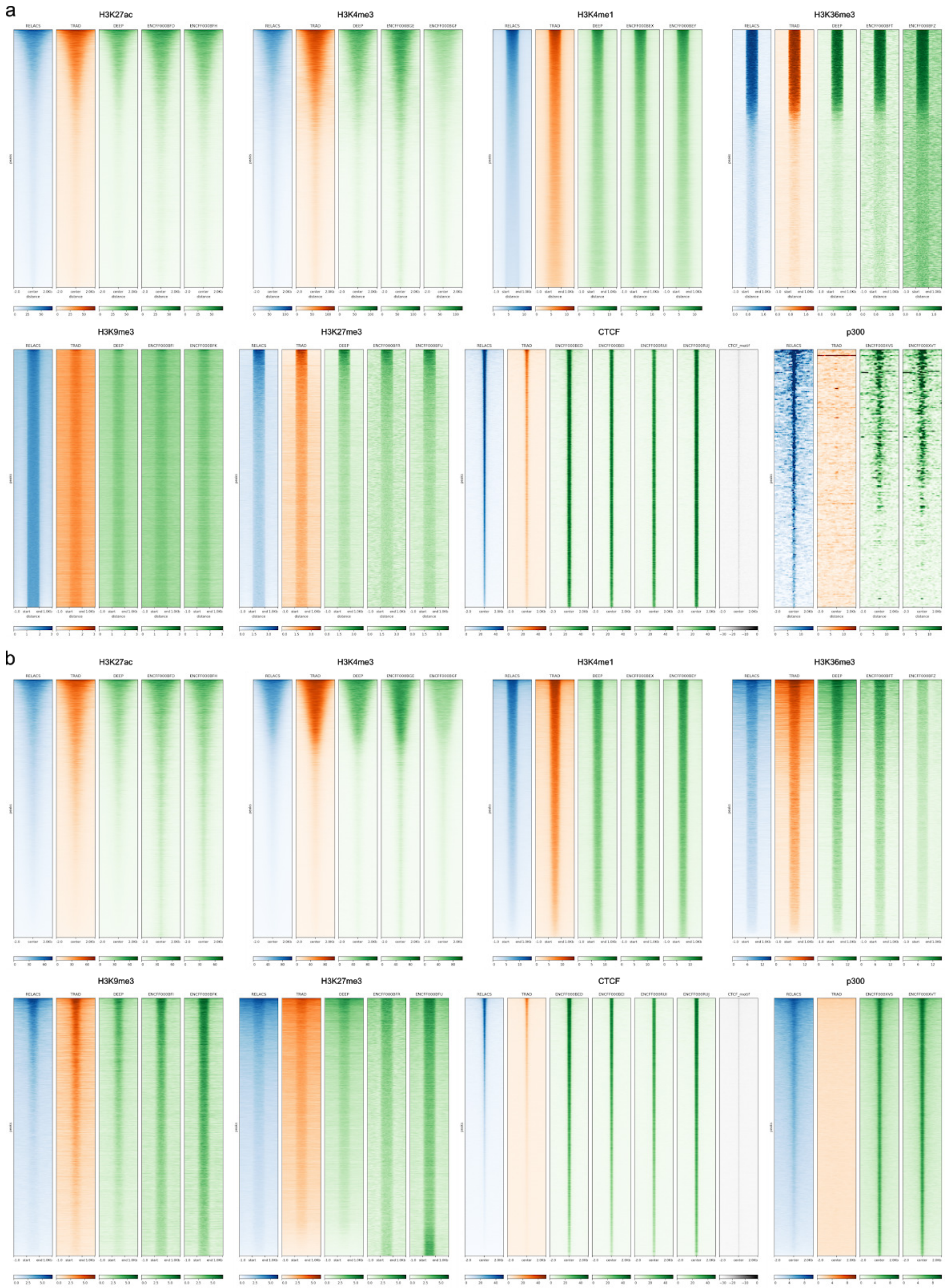
a
H3K27ac
H3K4me3
H3K4me1
H3K36me3
H3K9me3
H3K27me3
CTCF
p300
b
H3K27ac
H3K4me3
H3K4me1
H3K36me3
H3K9me3
H3K27me3
CTCF
p300

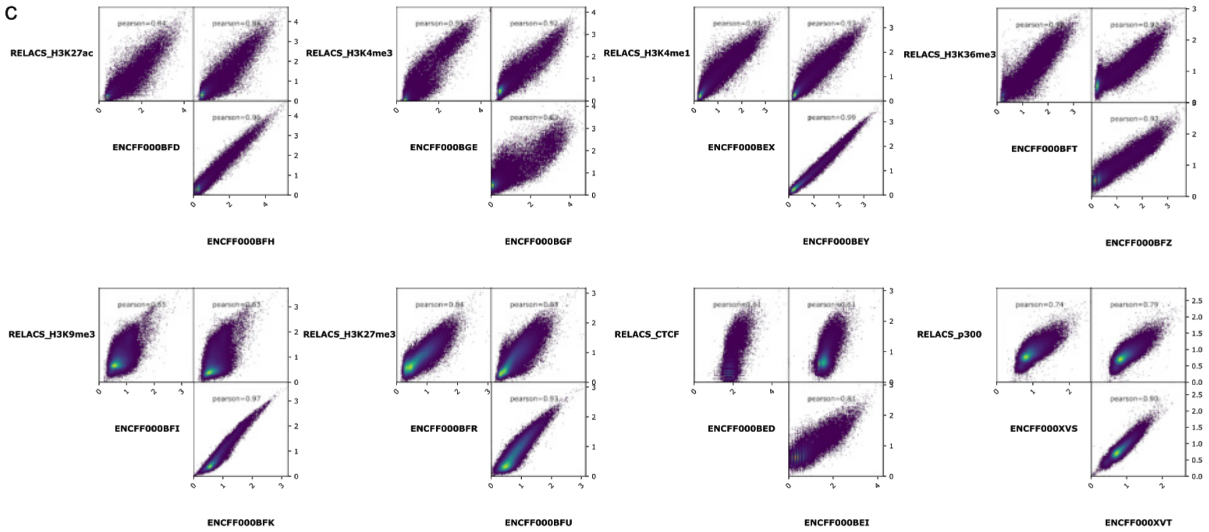

Supplementary Figure 7: Concordance of ChIP-seq signals from RELACS with ENCODE data. Here we show the concordance of all marks investigated in this studies **(a)** around peak regions defined by RELACS data (H3K27ac: 49856 regions; H3K4me3: 43072; H3K4me1: In 115,127; H3K36me3: 13,032; H3K9me3: 86,766; H3K27me3: 17,597; CTCF: 16,092; p300: 229) and **(b)** around peak regions defined by ENCODE (H3K27ac: 44,172 regions; H3K4me3: 48,113; H3K4me1: 52,166; H3K36me3: 13,320; H3K9me3: 17,539; H3K27me3: 27,889; CTCF: 73,888; p300: 36,421). For sharp marks (H3K4me3, H3K27ac, CTCF, p300) the plots are centered at the peak and a flanking regions of 2kb are added (using deepTools computeMatrix reference-point) For broad marks (H3K4me1, H3K36me3, H3K27me3, H3K9me3) were scaled to 1kb and an unscaled flanking regions of 1kb was added (computeMatrix scale-regions). The origin of the various samples is colour-coded as follows: blue=RELACS, orange=Traditional (this work), green=external data from IHEC/DEEP and ENCODE, grey=predicted motif scores (for CTCF only).

(c) shows a panel of scatter plots comparing the merged RELACS signal from all marks in this study with two corresponding replicates from the ENCODE project.

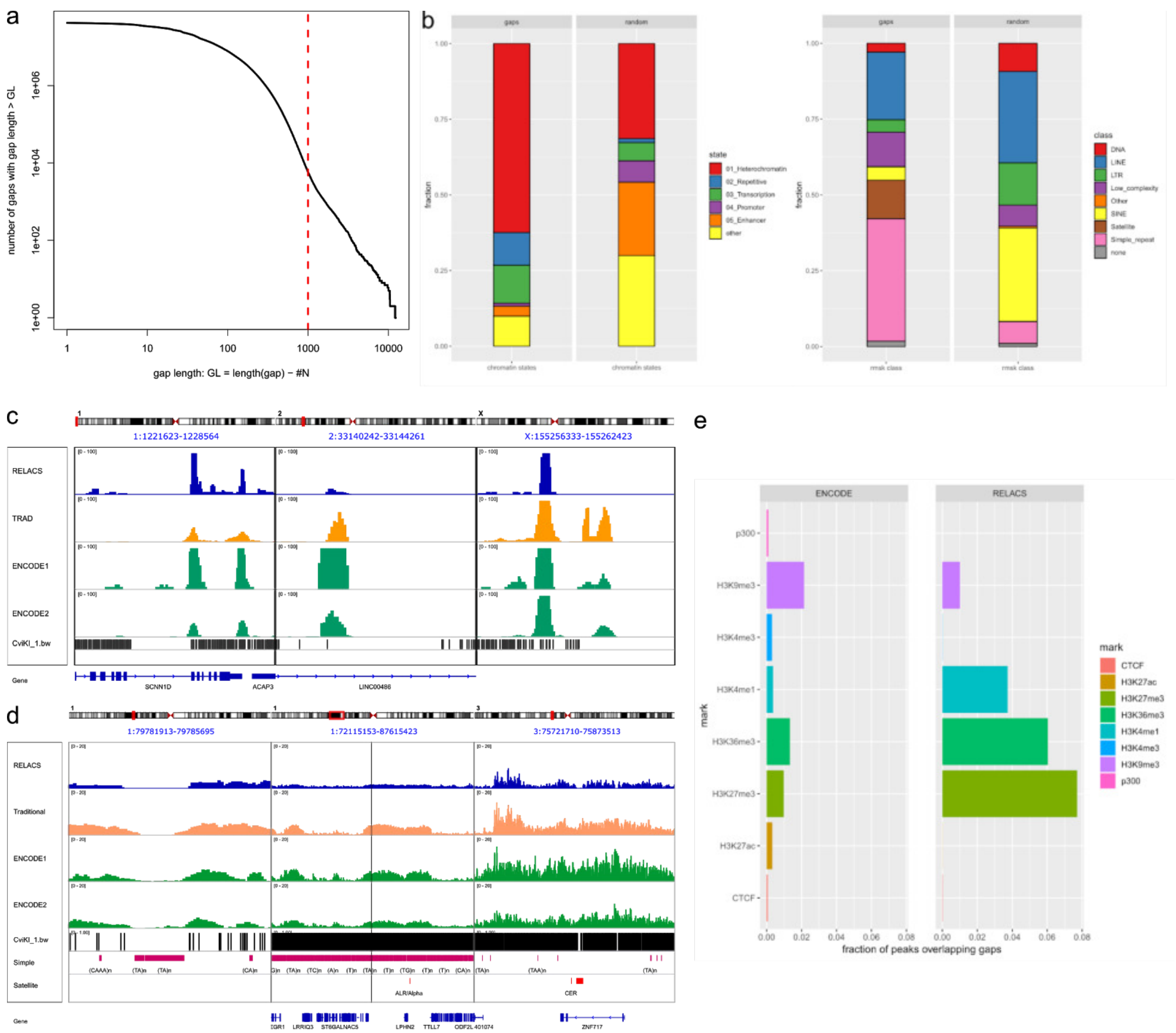

Supplementary Figure 8: Analysis of Restriction Gaps. (a) Here we show the distribution of gap length between restriction sites in the human genome, hs37hd5. We observe 6088 gaps with distances larger than 1000 bp which amounts to 0.29% of the genome. **(b)** Annotating these regions according to chromatin features with which they overlap, we find that large gaps preferentially reside in heterochromatin and repetitive regions - such as simple repeats, satellite and low complexity regions. **(c+d)** To illustrate possible consequences of gaps we show selected loci for CTCF and H3K9me3. The common color code for the tracks is as follows: blue=RELACS, orange=Traditional, green=ENCODE, black=restriction sites. **(c)** The locus on chromosome 1 shows a low complexity locus without restriction sites in an intron of gene SCNND1. The RELACS signal of an adjacent CTCF binding site is not affected,

since there is sufficient support from nearby fragments. The LINC00486 locus shows a strong reduction of CTCF signal from RELACS as a result of a low complexity region and a large RS-gaps. For the telomeric region of chromosome X a large fragment of CTCF signal is lost, but given the extensive low complexity region and possible copy number effects it is unclear whether this signal is genuine. This region would normally be blacklisted. **(d)** As illustrated above for the sharp mark CTCF, also H3K9me3 can be affected by gaps. The right locus shows a low complexity region (TA)n (red track) which does not contain any RS. Such regions are challenging for all studies because of low mappability which can be seen by a reduced coverage. The locus on the middle presents a much larger region (~15Mb) centered around the same gap. It is irrelevant on the scale of this large repressive domain. Similarly, the gap around the satellite repeat on the right locus (CER) is embedded in a much larger domain of H3K9me3 enrichment. **(e)** Summarizing the observations for all marks, we find that only 2% of annotated H3K9me3 ENCODE peaks overlap with larger gaps, while for sharp marks it is often less than 1%. Notice that even peaks called from the RELACS data can overlap with large gaps, if they have sufficients fragments from surrounding regions (as shown for the left locus of **(c)**).

a

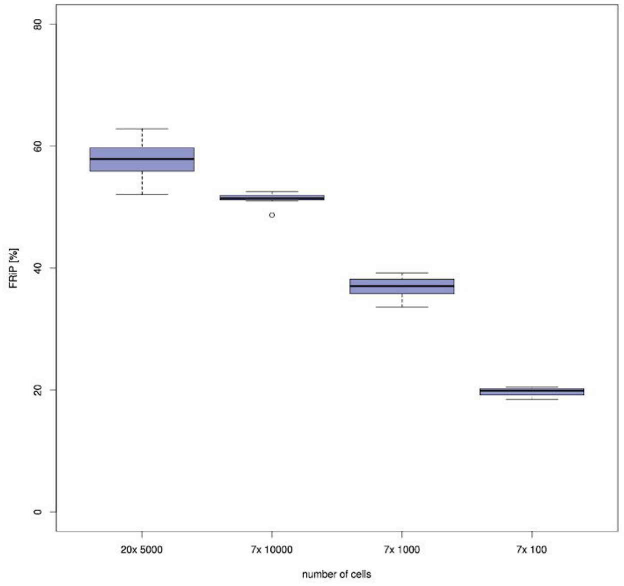

b

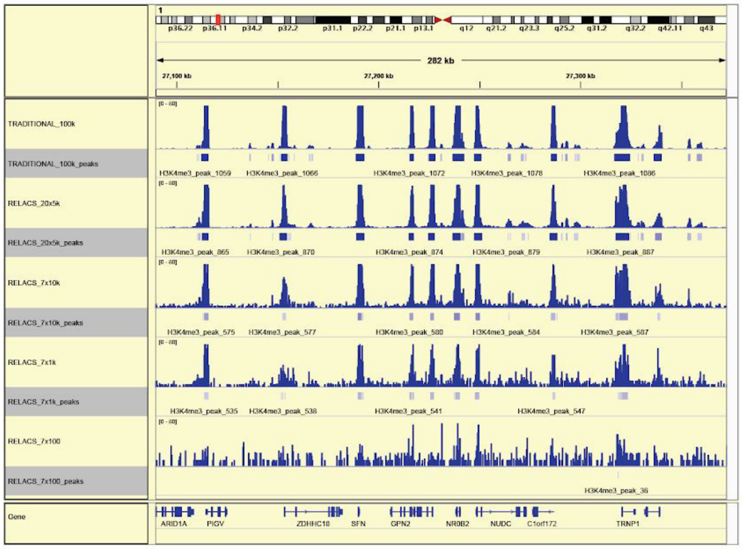

Supplementary Figure 9. ChIP-enrichments from low cell numbers. (a) Using ENCODE annotated peaks as reference, we calculated the fraction of reads that overlap with peaks (FRiP score). Shown are the distributions of FRiP scores for each settings of 20x5000, 7x10000, 7x1000 and 7x100 cells, where the first factor refers to the number of barcodes used in parallel for each ChIP (H3K4me3). **(b)** A snapshot from the IGV browser illustrates the high level of concordance between the signals and peaks for different setups. The first track is derived from data that was generated with 100000 cells and the traditional protocols. The following tracks are for RELACS with reducing number of cells: 20x 5000, 7x 10000, 7x 1000 and 7x 100 cells.

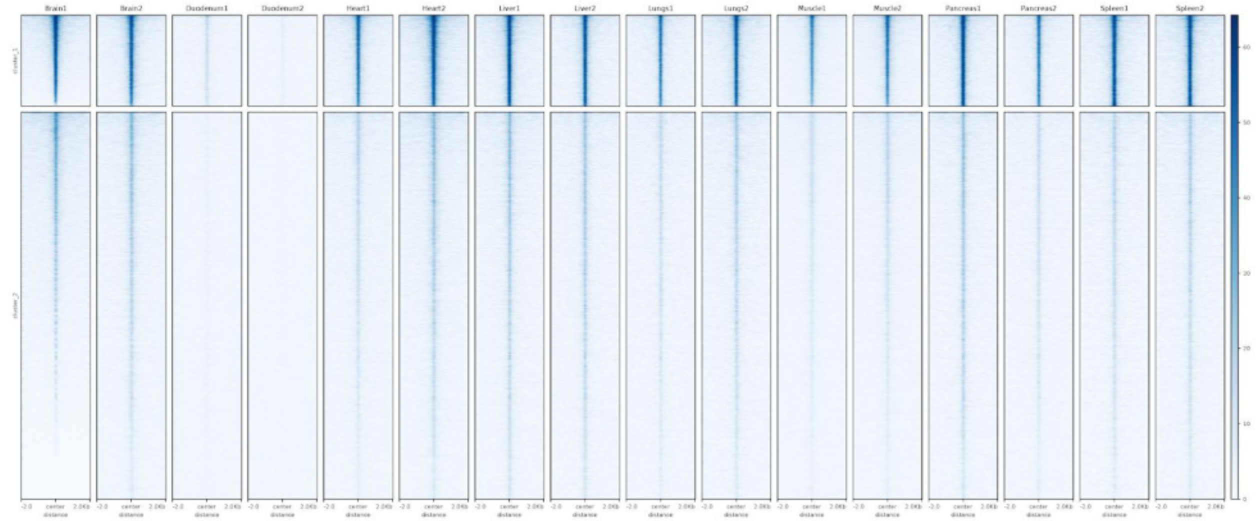

Supplementary Figure 10: Mouse CTCF data

Here we show consistent RELACS signals for CTCF around consensus peaks that have been previously defined for 18 different mouse cell types (see Methods). All tissues in our study (Brain, Duodenum, Heart, Liver, Lungs, Muscle, Pancreas, Spleen) were studied with two biological replicates from male littermates (25000 cells per tissue). The CTCF data is weaker for Duodenum (likely because of delayed fixation), but one can still observe support for the strongest CTCF peaks.

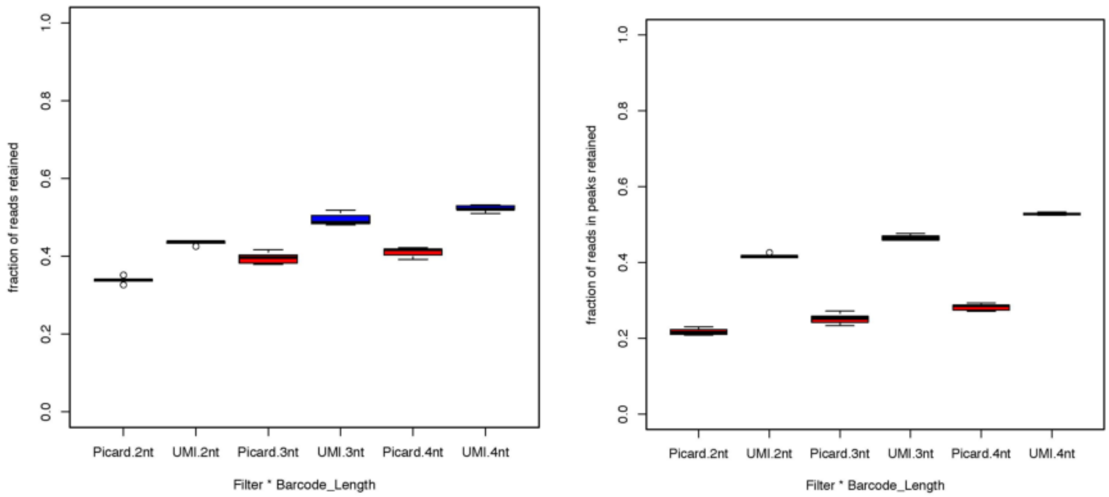

Supplementary Figure 11: UMI for improved duplicate filtering

As an outlook and further enhancement of the RELACS methodology we have modified 5 of the 20 barcodes from our main study and inserted 2, 3 or 4 random nucleotides (unique molecular identifiers) on both ends of the fragment. This allows us to retain a larger fraction of reads, that would otherwise be removed as possible "PCR duplicates" (by tools such as Picard). To illustrate this potential, we analyzed a CTCF sample from 30000 HepG2 cells with with high duplication rate (>50% at sequencing depth of around 10M reads per barcode). **(a)** Globally we see a 12% increase in the fraction of retained reads (50% vs 38%) when filtering only identical UMI-barcodes from the sequence (using UMItools). **(b)** In signal regions (CTCF peaks) the fraction of retained reads almost doubles (47% vs 25%).

a

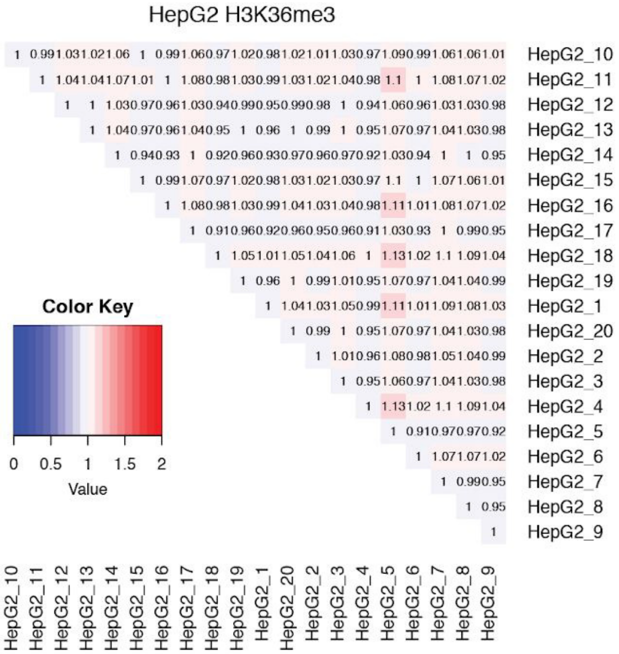

b

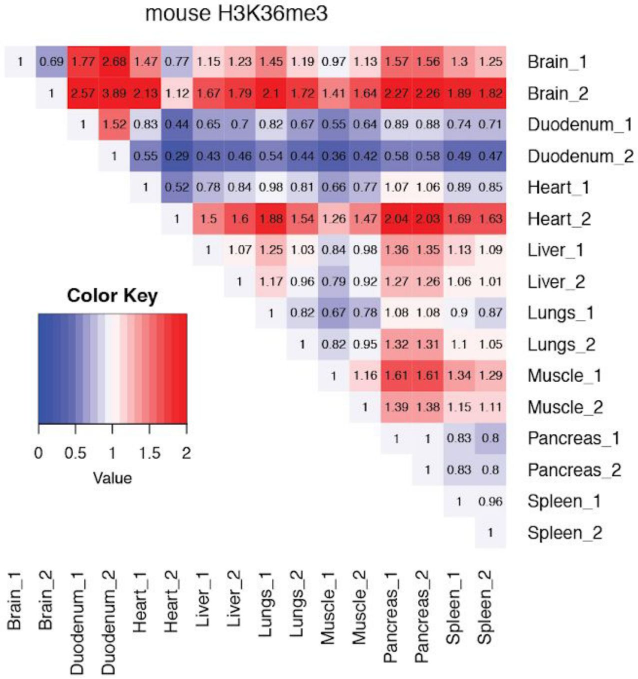

Supplementary Figure 12. Quantitative ChIP normalization. Here we calculate all pairwise changes between ChIPs as quantified by the double-ratio of (ChIP_1/Input_1)/(ChIP_2/Input_2). **(a)** H3K36me3 ChIP with 20 barcodes for the HepG2 cell line. As expected for technical replicates, all ratios are around 1 within 10%. **(b)** H3K36me3 ChIP for 16 different mouse tissues. Large observed changes point to overall quantitative differences between different samples.

Supplementary Tables

Supplementary Table 1. Comparison of different high-throughput ChIP-seq protocols. The table shows a summary and critical points of current high-throughput ChIP-seq procedures.

Supplementary Table 2. Sequence of barcoded adaptors. The table reports the sequences of barcoded adaptors. All of them have been used for cell line experiments. A subset of them has been used for mouse experiment (shown in main **Fig. 4**). The correspondence of barcode and related mouse tissue is indicated.

	High-throughput based on multiplexed ChIP						High-throughput based on robotic handling		
	Enzymatic				Sonication		Sonication		
	RELACS	Bar-ChIP	Mint-ChIP	combinatorial-iChIP	iChIP	co-ChIP	HT-ChIP	AHT-ChIP	R-ChIP
Cell number (input range for chromatin extraction)	10,000-1,000,0000	>1,000,000 (50 µg chromatin)	500-100,000	10,000,000	10,000-20,000	10,000,000-20,000,000	>10,000,000 (per experiment)	>10,000,000 (per experiment)	>50,000,000 (per experiment)
Sample number per experiment/run	flexible (1 to n)	flexible (1 to n)	flexible (1 to n)	flexible (1 to n)	flexible (1 to n)	flexible (1 to n)	max 96	max 96	max 96
Cell types tested	primary tissues (brain, liver, pancreas, skeletal muscle, heart, spleen, lung, duodenum), cell lines	yeast	cell lines, bone marrow hematopoietic stem cells	yeast	primary hematopoietic cells (several stages of development), bone marrow dendritic cells	primary tissues (liver, kidney, brain, lung), bone marrow dendritic cells, cell lines	cultured bone marrow dendritic cells	primary tissues (liver), cell lines	cell lines
Epitopes tested	histone modifications (active and repressive), transcription factors	histone modifications (active)	histone modifications (active and repressive)	histone modifications (active)	histone modifications (active), transcription factors	histone modifications (active and repressive), transcription factors	histone modifications (active and repressive), transcription factors	histone modifications (active), transcription factors	histone modifications (active), transcription factors
Time	3 days	not reported	not reported	not reported	not reported	not reported	3 days	400 ChIP in 5 days	not reported
Automation	yes (ChIP), not mandatory	no	no	no	no	no	yes (DNA purification, library)	yes (from ChIP to library)	yes (ChIP)
Fixation	yes	yes	no	yes	yes	yes	yes	yes	yes (30min 37 C)
Chromatin fragmentation method	restriction enzymes	MNase	MNase	MNase	sonication	sonication	sonication	sonication	sonication
Chromatin barcoding	yes	yes	yes	yes	yes	yes	no	no	no
Re-ChIP for barcoding needed?	no	no	no	yes	yes	yes	no chromatin barcoding	no chromatin barcoding	no chromatin barcoding
Minimal number of cells per IP	>1,000 (histones), >10,000 (TF)	>100,000	>5000	not reported	>5000	not reported; 10,000,000 (for the first ChIP, when against TFs)	10,000-100,000 (histones), 100,000-10,000,000 (TFs)	>1,000,000	not reported
Minimal number of cells per IP tested in the study	100	> 100,000	500 (+ chromatin carrier)	not reported	500	not reported	10,000	1,000	not reported
Linear amplification of ChIP-DNA	no	no	yes	no	no	no	no	no	no
Protocol description	Cell fixation, nuclei extraction, intra-nuclei chormatin digestion (>70% at 100-1000 bp) and barcoding. Pooling of barcoded nuclei, lysis, ChIP on chromatin pool. DNA purification and PCR amplification.	Cell fixation, lysis, MNase chromatin fragmentation (to ~80% mononucleosomes). Chromatin barcoding in solution. Pooling of chromatin for multiplexed ChIP. Purification of ChIP DNA, PCR amplification.	Cell fixation, lysis, MNase chromatin fragmentation at two optimized MNase concentration per sample (low/high, to 1-5 nucleosomes). Chromatin barcoding in solution. Pooling of samples at low and high MNase concentration into two separated pools for combined ChIP (with chromatin carrier). Purification of ChIP DNA, in-vitro transcription, reverse transcription, PCR amplification of cDNA.	Similar to iChIP/co-ChIP, it is used to measure co-occurence of modifications on the same genomic regions at nucleosome resolution. Cell fixation, lysis, MNase chromatin digestion (to ~80% mononucleosomes), first ChIP with antibody of interest (histone modifications). Chromatin is barcoded, released from beads and pooled prior multiplexed ChIP (second ChIP using antibody of interest). Purification of ChIP DNA, PCR amplification.	Cell fixation, sonication, anti-H3 ChIP (first ChIP) and barcoding of chromatin immobilized to beads. Barcoded chromatin is released and pooled for the second combined ChIP using antibody of interest. Purification of ChIP DNA, PCR amplification.	Similar to iChIP, it is used to measure co-occurence of modifications on the same genomic regions. Cell fixation, sonication, first ChIP with antibody of interest (histone modifications/TFs, not H3). Chromatin is barcoded, released from beads and pooled prior combined ChIP (second ChIP using antibody of interest). Purification of ChIP DNA, PCR amplification.	Cell fixation, chromatin extraction and sonication. ChIP (antibody incubation, sequestration of immunocomplexes with beads, beads washes, reverse crosslink) is carried out manually in 96-well plates. DNA purification and library preparation is automated using the Bravo Agilent liquid handler.	Cell fixation, chromatin extraction and sonication. Chromatin immunoprecipitation, beads washes, reverse crosslink, DNA purification and library construction is carried out automatically with user interventions (Bravo Agilent for ChIP and Beckman Fx liquid handler for library preparation).	Cell fixation, chromatin extraction and sonication. Chromatin immunoprecipitation, beads washes, reverse crosslink are automated steps (Tecan Freedom EVO 200 liquid handler). Manual DNA purification and library construction.
Reference	This study	Chabbert, C. D. et al. A high-throughput ChIP-Seq for large-scale chromatin studies. Mol. Syst. Biol. 11, 777 (2015).	van Galen, P. et al. A Multiplexed System for Quantitative Comparisons of Chromatin Landscapes. Mol. Cell 61, 170–180 (2016).	Sadeh, R., Launer-Wachs, R., Wandel, H., Rahat, A. & Friedman, N. Elucidating Combinatorial Chromatin States at Single-Nucleosome Resolution. Mol. Cell 63, 1080–1088 (2016).	Lara-Astiaso, D. et al. Chromatin state dynamics during blood formation. Science 1–10 (2014).	Weiner, A. et al. Co-ChIP enables genome-wide mapping of histone mark co-occurrence at single-molecule resolution. Nat. Biotechnol. 34, 953–961 (2016).	Garber, M. et al. A high-throughput chromatin immunoprecipitation approach reveals principles of dynamic gene regulation in mammals. Mol. Cell 47, 810–822 (2012).	Aldridge, S. et al. AHT-ChIP-seq: a completely automated robotic protocol for high-throughput chromatin immunoprecipitation. Genome Biol. 14, R124 (2013).	Gasper, W. C. et al. Fully automated high-throughput chromatin immunoprecipitation for ChIP-seq: identifying ChIP-quality p300 monoclonal antibodies. Sci. Rep. 4, 5152 (2014).

Supplementary Table 1. Comparison of different high-throughput ChIP-seq protocols. The table shows a summary and critical points of current high-throughput ChIP-seq procedures.

Name	Sequence	Mouse tissues experiments
Bar_1	/5Phos/ATAATATCAGATCGGAAGAGCACACGTCTGAACTCCAGTC/ideoxyU/ACACTCTTTCCCTACACGACGCTCTTCCGATCTGATATTAT*T	Liver_rep1
Bar_2	/5Phos/AAAGCGGCAGATCGGAAGAGCACACGTCTGAACTCCAGTC/ideoxyU/ACACTCTTTCCCTACACGACGCTCTTCCGATCTGCCGCTTT*T	Liver_rep2
Bar_3	/5Phos/GCGAATGCAGATCGGAAGAGCACACGTCTGAACTCCAGTC/ideoxyU/ACACTCTTTCCCTACACGACGCTCTTCCGATCTGCATTCGC*T	Brain_rep1
Bar_4	/5Phos/CTGGCGGCAGATCGGAAGAGCACACGTCTGAACTCCAGTC/ideoxyU/ACACTCTTTCCCTACACGACGCTCTTCCGATCTGCCGCCAG*T	Brain_rep2
Bar_5	/5Phos/CCAACTCGAGATCGGAAGAGCACACGTCTGAACTCCAGTC/ideoxyU/ACACTCTTTCCCTACACGACGCTCTTCCGATCTCGAGTTGG*T	Pancreas_rep1
Bar_6	/5Phos/GAAAATTTAGATCGGAAGAGCACACGTCTGAACTCCAGTC/ideoxyU/ACACTCTTTCCCTACACGACGCTCTTCCGATCTAAATTTTC*T	Pancreas_rep2
Bar_7	/5Phos/CAAACGCTAGATCGGAAGAGCACACGTCTGAACTCCAGTC/ideoxyU/ACACTCTTTCCCTACACGACGCTCTTCCGATCTAGCGTTTG*T	Heart_rep1
Bar_8	/5Phos/CTGACGCCAGATCGGAAGAGCACACGTCTGAACTCCAGTC/ideoxyU/ACACTCTTTCCCTACACGACGCTCTTCCGATCTGGCGTCAG*T	Heart_rep2
Bar_9	/5Phos/CCAGTATGAGATCGGAAGAGCACACGTCTGAACTCCAGTC/ideoxyU/ACACTCTTTCCCTACACGACGCTCTTCCGATCTCATACTGG*T	Spleen_rep1
Bar_10	/5Phos/AAAATGCTAGATCGGAAGAGCACACGTCTGAACTCCAGTC/ideoxyU/ACACTCTTTCCCTACACGACGCTCTTCCGATCTAGCATTTT*T	Spleen_rep2
Bar_11	/5Phos/GTGACTAGAGATCGGAAGAGCACACGTCTGAACTCCAGTC/ideoxyU/ACACTCTTTCCCTACACGACGCTCTTCCGATCTCTAGTCAC*T	Lungs_rep1
Bar_12	/5Phos/ATGACAGAAGATCGGAAGAGCACACGTCTGAACTCCAGTC/ideoxyU/ACACTCTTTCCCTACACGACGCTCTTCCGATCTTCTGTCAT*T	Lungs_rep2
Bar_13	/5Phos/GGAACAGCAGATCGGAAGAGCACACGTCTGAACTCCAGTC/ideoxyU/ACACTCTTTCCCTACACGACGCTCTTCCGATCTGCTGTTCC*T	Skeletal muscle_rep1
Bar_14	/5Phos/CTGGCTCTAGATCGGAAGAGCACACGTCTGAACTCCAGTC/ideoxyU/ACACTCTTTCCCTACACGACGCTCTTCCGATCTAGAGCCAG*T	Skeletal muscle_rep2
Bar_15	/5Phos/AGGGACTGAGATCGGAAGAGCACACGTCTGAACTCCAGTC/ideoxyU/ACACTCTTTCCCTACACGACGCTCTTCCGATCTCAGTCCCT*T	Duodenum_rep1
Bar_16	/5Phos/CAGGTAATAGATCGGAAGAGCACACGTCTGAACTCCAGTC/ideoxyU/ACACTCTTTCCCTACACGACGCTCTTCCGATCTATTACCTG*T	Duodenum_rep2
Bar_17	/5Phos/AAAACACTAGATCGGAAGAGCACACGTCTGAACTCCAGTC/ideoxyU/ACACTCTTTCCCTACACGACGCTCTTCCGATCTAGTGTTTT*T	
Bar_18	/5Phos/AAGGGATGAGATCGGAAGAGCACACGTCTGAACTCCAGTC/ideoxyU/ACACTCTTTCCCTACACGACGCTCTTCCGATCTCATCCCTT*T	
Bar_19	/5Phos/GCGAAGCAAGATCGGAAGAGCACACGTCTGAACTCCAGTC/ideoxyU/ACACTCTTTCCCTACACGACGCTCTTCCGATCTTGCTTCGC*T	
Bar_20	/5Phos/GGGAGACGAGATCGGAAGAGCACACGTCTGAACTCCAGTC/ideoxyU/ACACTCTTTCCCTACACGACGCTCTTCCGATCTCGTCTCCC*T	
Bar_2_UMI_2nt	/5Phos/AAAGCGGCNNAGATCGGAAGAGCACACGTCTGAACTCCAGTC/ideoxyU/ACACTCTTTCCCTACACGACGCTCTTCCGATCTNNGCCGCTTT*T	
Bar_5_UMI_2nt	/5Phos/CCAACTCGNNAGATCGGAAGAGCACACGTCTGAACTCCAGTC/ideoxyU/ACACTCTTTCCCTACACGACGCTCTTCCGATCTNNCGAGTTGG*T	
Bar_16_UMI_2nt	/5Phos/CAGGTAATNNAGATCGGAAGAGCACACGTCTGAACTCCAGTC/ideoxyU/ACACTCTTTCCCTACACGACGCTCTTCCGATCTNNATTACCTG*T	
Bar_18_UMI_2nt	/5Phos/AAGGGATGNNAGATCGGAAGAGCACACGTCTGAACTCCAGTC/ideoxyU/ACACTCTTTCCCTACACGACGCTCTTCCGATCTNNCATCCCTT*T	
Bar_20_UMI_2nt	/5Phos/GGGAGACGNNAGATCGGAAGAGCACACGTCTGAACTCCAGTC/ideoxyU/ACACTCTTTCCCTACACGACGCTCTTCCGATCTNNCGTCTCCC*T	
Bar_2_UMI_3nt	/5Phos/AAAGCGGCNNNAGATCGGAAGAGCACACGTCTGAACTCCAGTC/ideoxyU/ACACTCTTTCCCTACACGACGCTCTTCCGATCTNNNGCCGCTTT*T	
Bar_5_UMI_3nt	/5Phos/CCAACTCGNNNAGATCGGAAGAGCACACGTCTGAACTCCAGTC/ideoxyU/ACACTCTTTCCCTACACGACGCTCTTCCGATCTNNNCGAGTTGG*T	
Bar_16_UMI_3nt	/5Phos/CAGGTAATNNNAGATCGGAAGAGCACACGTCTGAACTCCAGTC/ideoxyU/ACACTCTTTCCCTACACGACGCTCTTCCGATCTNNNATTACCTG*T	
Bar_18_UMI_3nt	/5Phos/AAGGGATGNNNAGATCGGAAGAGCACACGTCTGAACTCCAGTC/ideoxyU/ACACTCTTTCCCTACACGACGCTCTTCCGATCTNNNCATCCCTT*T	
Bar_20_UMI_3nt	/5Phos/GGGAGACGNNNAGATCGGAAGAGCACACGTCTGAACTCCAGTC/ideoxyU/ACACTCTTTCCCTACACGACGCTCTTCCGATCTNNNCGTCTCCC*T	
Bar_2_UMI_4nt	/5Phos/AAAGCGGCNNNNAGATCGGAAGAGCACACGTCTGAACTCCAGTC/ideoxyU/ACACTCTTTCCCTACACGACGCTCTTCCGATCTNNNNGCCGCTTT*T	
Bar_5_UMI_4nt	/5Phos/CCAACTCGNNNNAGATCGGAAGAGCACACGTCTGAACTCCAGTC/ideoxyU/ACACTCTTTCCCTACACGACGCTCTTCCGATCTNNNNCGAGTTGG*T	
Bar_16_UMI_4nt	/5Phos/CAGGTAATNNNNAGATCGGAAGAGCACACGTCTGAACTCCAGTC/ideoxyU/ACACTCTTTCCCTACACGACGCTCTTCCGATCTNNNNATTACCTG*T	
Bar_18_UMI_4nt	/5Phos/AAGGGATGNNNNAGATCGGAAGAGCACACGTCTGAACTCCAGTC/ideoxyU/ACACTCTTTCCCTACACGACGCTCTTCCGATCTNNNNCATCCCTT*T	
Bar_20_UMI_4nt	/5Phos/GGGAGACGNNNNAGATCGGAAGAGCACACGTCTGAACTCCAGTC/ideoxyU/ACACTCTTTCCCTACACGACGCTCTTCCGATCTNNNNCGTCTCCC*T	

Supplementary Table 2. Sequence of barcoded adaptors.
The table reports the sequences of barcoded adaptors. All of them have been used for cell line experiments. A subset of them has been used for mouse experiment (shown in main Fig. 4). The correspondence of barcode and related mouse tissue is indicated.
