# Supplementary material for: RELACS nuclei barcoding enables high-throughput ChIP-seq
Source: Commun Biol. 2018 Dec 5;1:214. doi: 10.1038/s42003-018-0219-z (PMC6281648; doi:10.1038/s42003-018-0219-z)
Supplement: Supplementary file 2 — Description of Additional Supplementary Files [file 42003_2018_219_MOESM2_ESM.docx]

**Description of Additional Supplementary Files**

**File Name**: Supplementary Data 1

**Description**: HepG2 RELACS. QC metrics for HepG2 data obtained with RELACS protocol with 20 barcodes (20 x 5000 cells).

**File Name**: Supplementary Data 2

**Description**: HepG2 traditional. QC metrics for HepG2 data obtained with traditional ChIP-seq protocol (1x 100000 cells)

**File Name**: Supplementary Data 3

**Description**: Low cell number RELACS. QC metrics for HepG2 data obtained with RELACS protocol with 7 barcodes for small cell numbers (100,1000,10000 cells).

**File Name**: Supplementary Data 4

**Description**: Mouse RELACS. QC metrics for data from 16 mouse tissues obtained with RELACS protocol with 16 barcodes (16 x 25000 cells).
