# Supplementary material for: RELACS nuclei barcoding enables high-throughput ChIP-seq
Source: Commun Biol. 2018 Dec 5;1:214. doi: 10.1038/s42003-018-0219-z (PMC6281648; doi:10.1038/s42003-018-0219-z)
Supplement: Supplementary file 5 — Supplementary Data 3 [file 42003_2018_219_MOESM5_ESM.html]

MultiQC Report


# Toggle navigation v1.3

Loading report..

- General Stats
- deepTools
- Bowtie 2

Toolbox

### MultiQC Toolbox

#### Apply Highlight Samples

+

Regex mode off
help
 Clear

#### Apply Rename Samples

+

Click here for bulk input.

Paste two columns of a tab-delimited table here (eg. from Excel).

First column should be the old name, second column the new name.

Add

Regex mode off
help
 Clear

#### Apply Show / Hide Samples

Hide matching samples

Show only matching samples

+

Regex mode off
help
 Clear

#### Export Plots

- Images
- Data

px

px

Aspect ratio

PNG
JPEG
SVG

Plot scaling

X

Download the raw data used to create the plots in this report below:

Format:

Tab-separated
Comma-separated
JSON

Note that additional data was saved in `multiqc_data` when this report was generated.

---

##### Choose Plots

 All
 None

bowtie2\_pe\_plot

tableScatterPlot

bowtie2\_pe\_plot

tableScatterPlot

---


   Download Plot Images

If you use plots from MultiQC in a publication or presentation, please cite:

> **MultiQC: Summarize analysis results for multiple tools and samples in a single report**  
> *Philip Ewels, Måns Magnusson, Sverker Lundin and Max Käller*  
> Bioinformatics (2016)  
> doi: 10.1093/bioinformatics/btw354  
> PMID: 27312411

#### Save Settings

You can save the toolbox settings for this report to the browser.

 Save


---

#### Load Settings

Choose a saved report profile from the dropdown box below:

[ select ]

Load
 Delete
 Set default
 Clear default

#### About MultiQC

This report was generated using MultiQC, version 1.3

You can see a YouTube video describing how to use MultiQC reports here:
https://youtu.be/qPbIlO\_KWN0

For more information about MultiQC, including other videos and
extensive documentation, please visit http://multiqc.info

You can report bugs, suggest improvements and find the source code for MultiQC on GitHub:
https://github.com/ewels/MultiQC

MultiQC is published in Bioinformatics:

> **MultiQC: Summarize analysis results for multiple tools and samples in a single report**  
> *Philip Ewels, Måns Magnusson, Sverker Lundin and Max Käller*  
> Bioinformatics (2016)  
> doi: 10.1093/bioinformatics/btw354  
> PMID: 27312411

# 

A modular tool to aggregate results from bioinformatics analyses across many samples into a single report.

#### JavaScript Disabled

MultiQC reports use JavaScript for plots and toolbox functions. It looks like
you have JavaScript disabled in your web browser. Please note that many of the report
functions will not work as intended.

Loading report..

---

## General Statistics

 Copy table

 Sort by highlight
Showing 84/84 rows and 1/1 columns.

| Sample Name | % Aligned |
| --- | --- |
| RELACS\_CTCF\_10000\_Rep2\_15min\_10.Bowtie2 | 99.7% |
| RELACS\_CTCF\_10000\_Rep2\_15min\_11.Bowtie2 | 99.7% |
| RELACS\_CTCF\_10000\_Rep2\_15min\_12.Bowtie2 | 99.3% |
| RELACS\_CTCF\_10000\_Rep2\_15min\_13.Bowtie2 | 99.5% |
| RELACS\_CTCF\_10000\_Rep2\_15min\_14.Bowtie2 | 99.6% |
| RELACS\_CTCF\_10000\_Rep2\_15min\_8.Bowtie2 | 99.3% |
| RELACS\_CTCF\_10000\_Rep2\_15min\_9.Bowtie2 | 99.7% |
| RELACS\_CTCF\_1000\_Rep2\_15min\_10.Bowtie2 | 99.6% |
| RELACS\_CTCF\_1000\_Rep2\_15min\_11.Bowtie2 | 99.6% |
| RELACS\_CTCF\_1000\_Rep2\_15min\_12.Bowtie2 | 99.4% |
| RELACS\_CTCF\_1000\_Rep2\_15min\_13.Bowtie2 | 99.5% |
| RELACS\_CTCF\_1000\_Rep2\_15min\_14.Bowtie2 | 99.5% |
| RELACS\_CTCF\_1000\_Rep2\_15min\_8.Bowtie2 | 99.2% |
| RELACS\_CTCF\_1000\_Rep2\_15min\_9.Bowtie2 | 99.6% |
| RELACS\_CTCF\_100\_Rep2\_15min\_10.Bowtie2 | 99.6% |
| RELACS\_CTCF\_100\_Rep2\_15min\_11.Bowtie2 | 99.5% |
| RELACS\_CTCF\_100\_Rep2\_15min\_12.Bowtie2 | 99.4% |
| RELACS\_CTCF\_100\_Rep2\_15min\_13.Bowtie2 | 99.4% |
| RELACS\_CTCF\_100\_Rep2\_15min\_14.Bowtie2 | 99.5% |
| RELACS\_CTCF\_100\_Rep2\_15min\_8.Bowtie2 | 99.1% |
| RELACS\_CTCF\_100\_Rep2\_15min\_9.Bowtie2 | 99.6% |
| RELACS\_H3K27me3\_10000\_Rep2\_15min\_10.Bowtie2 | 99.3% |
| RELACS\_H3K27me3\_10000\_Rep2\_15min\_11.Bowtie2 | 99.3% |
| RELACS\_H3K27me3\_10000\_Rep2\_15min\_12.Bowtie2 | 99.3% |
| RELACS\_H3K27me3\_10000\_Rep2\_15min\_13.Bowtie2 | 99.2% |
| RELACS\_H3K27me3\_10000\_Rep2\_15min\_14.Bowtie2 | 99.2% |
| RELACS\_H3K27me3\_10000\_Rep2\_15min\_8.Bowtie2 | 99.0% |
| RELACS\_H3K27me3\_10000\_Rep2\_15min\_9.Bowtie2 | 99.4% |
| RELACS\_H3K27me3\_1000\_Rep2\_15min\_10.Bowtie2 | 99.5% |
| RELACS\_H3K27me3\_1000\_Rep2\_15min\_11.Bowtie2 | 99.5% |
| RELACS\_H3K27me3\_1000\_Rep2\_15min\_12.Bowtie2 | 99.5% |
| RELACS\_H3K27me3\_1000\_Rep2\_15min\_13.Bowtie2 | 99.3% |
| RELACS\_H3K27me3\_1000\_Rep2\_15min\_14.Bowtie2 | 99.4% |
| RELACS\_H3K27me3\_1000\_Rep2\_15min\_8.Bowtie2 | 99.1% |
| RELACS\_H3K27me3\_1000\_Rep2\_15min\_9.Bowtie2 | 99.5% |
| RELACS\_H3K27me3\_100\_Rep2\_15min\_10.Bowtie2 | 99.6% |
| RELACS\_H3K27me3\_100\_Rep2\_15min\_11.Bowtie2 | 99.6% |
| RELACS\_H3K27me3\_100\_Rep2\_15min\_12.Bowtie2 | 99.1% |
| RELACS\_H3K27me3\_100\_Rep2\_15min\_13.Bowtie2 | 99.5% |
| RELACS\_H3K27me3\_100\_Rep2\_15min\_14.Bowtie2 | 99.5% |
| RELACS\_H3K27me3\_100\_Rep2\_15min\_8.Bowtie2 | 99.2% |
| RELACS\_H3K27me3\_100\_Rep2\_15min\_9.Bowtie2 | 99.6% |
| RELACS\_H3K4me3\_10000\_Rep2\_15min\_10.Bowtie2 | 99.4% |
| RELACS\_H3K4me3\_10000\_Rep2\_15min\_11.Bowtie2 | 99.5% |
| RELACS\_H3K4me3\_10000\_Rep2\_15min\_12.Bowtie2 | 99.2% |
| RELACS\_H3K4me3\_10000\_Rep2\_15min\_13.Bowtie2 | 99.3% |
| RELACS\_H3K4me3\_10000\_Rep2\_15min\_14.Bowtie2 | 99.4% |
| RELACS\_H3K4me3\_10000\_Rep2\_15min\_8.Bowtie2 | 99.0% |
| RELACS\_H3K4me3\_10000\_Rep2\_15min\_9.Bowtie2 | 99.5% |
| RELACS\_H3K4me3\_1000\_Rep2\_15min\_10.Bowtie2 | 99.5% |
| RELACS\_H3K4me3\_1000\_Rep2\_15min\_11.Bowtie2 | 99.6% |
| RELACS\_H3K4me3\_1000\_Rep2\_15min\_12.Bowtie2 | 99.5% |
| RELACS\_H3K4me3\_1000\_Rep2\_15min\_13.Bowtie2 | 99.4% |
| RELACS\_H3K4me3\_1000\_Rep2\_15min\_14.Bowtie2 | 99.5% |
| RELACS\_H3K4me3\_1000\_Rep2\_15min\_8.Bowtie2 | 99.1% |
| RELACS\_H3K4me3\_1000\_Rep2\_15min\_9.Bowtie2 | 99.5% |
| RELACS\_H3K4me3\_100\_Rep2\_15min\_10.Bowtie2 | 99.5% |
| RELACS\_H3K4me3\_100\_Rep2\_15min\_11.Bowtie2 | 99.4% |
| RELACS\_H3K4me3\_100\_Rep2\_15min\_12.Bowtie2 | 99.3% |
| RELACS\_H3K4me3\_100\_Rep2\_15min\_13.Bowtie2 | 99.4% |
| RELACS\_H3K4me3\_100\_Rep2\_15min\_14.Bowtie2 | 99.4% |
| RELACS\_H3K4me3\_100\_Rep2\_15min\_8.Bowtie2 | 98.5% |
| RELACS\_H3K4me3\_100\_Rep2\_15min\_9.Bowtie2 | 99.5% |
| RELACS\_Input\_10000\_Rep2\_15min\_10.Bowtie2 | 99.6% |
| RELACS\_Input\_10000\_Rep2\_15min\_11.Bowtie2 | 99.6% |
| RELACS\_Input\_10000\_Rep2\_15min\_12.Bowtie2 | 99.5% |
| RELACS\_Input\_10000\_Rep2\_15min\_13.Bowtie2 | 99.5% |
| RELACS\_Input\_10000\_Rep2\_15min\_14.Bowtie2 | 99.5% |
| RELACS\_Input\_10000\_Rep2\_15min\_8.Bowtie2 | 99.3% |
| RELACS\_Input\_10000\_Rep2\_15min\_9.Bowtie2 | 99.6% |
| RELACS\_Input\_1000\_Rep2\_15min\_10.Bowtie2 | 99.6% |
| RELACS\_Input\_1000\_Rep2\_15min\_11.Bowtie2 | 99.6% |
| RELACS\_Input\_1000\_Rep2\_15min\_12.Bowtie2 | 99.6% |
| RELACS\_Input\_1000\_Rep2\_15min\_13.Bowtie2 | 99.4% |
| RELACS\_Input\_1000\_Rep2\_15min\_14.Bowtie2 | 99.5% |
| RELACS\_Input\_1000\_Rep2\_15min\_8.Bowtie2 | 99.2% |
| RELACS\_Input\_1000\_Rep2\_15min\_9.Bowtie2 | 99.6% |
| RELACS\_Input\_100\_Rep2\_15min\_10.Bowtie2 | 99.2% |
| RELACS\_Input\_100\_Rep2\_15min\_11.Bowtie2 | 98.9% |
| RELACS\_Input\_100\_Rep2\_15min\_12.Bowtie2 | 98.8% |
| RELACS\_Input\_100\_Rep2\_15min\_13.Bowtie2 | 99.0% |
| RELACS\_Input\_100\_Rep2\_15min\_14.Bowtie2 | 98.8% |
| RELACS\_Input\_100\_Rep2\_15min\_8.Bowtie2 | 98.4% |
| RELACS\_Input\_100\_Rep2\_15min\_9.Bowtie2 | 98.6% |

×

#### General Statistics: Columns

Uncheck the tick box to hide columns. Click and drag the handle on the left to change order.

Show All
Show None

| Sort | Visible | Group | Column | Description | ID | Scale |
| --- | --- | --- | --- | --- | --- | --- |
| || |  | Bowtie 2 | % Aligned | overall alignment rate | `overall_alignment_rate` | None |

Close

## deepTools

deepTools is a suite of tools to process and analyze deep sequencing data.

### Filtering metrics

Estimated percentages of alignments filtered independently for each setting in `estimateReadFiltering` . The tool was excuted with the following arguments: `--samFlagInclude 2 --samFlagExclude 1024 --ignoreDuplicates --minMappingQuality 1 --blackListFileName hg19_blacklist`

`hg19_blacklist`

Copy table

 Configure Columns

 Sort by highlight

 Plot
Showing 84/84 rows and 10/10 columns.

| Sample Name | M entries | % Aligned | % Tot. Filtered | % Blacklisted | % Missing Flags | % Forbidden Flags | % deepTools Dupes | % Duplication | % Singletons | % Strand Filtered |
| --- | --- | --- | --- | --- | --- | --- | --- | --- | --- | --- |
| RELACS\_CTCF\_10000\_Rep2\_15min\_10 | 1.3 | 99.6 | 36.2 | 3.5 | 10.7 | 39.9 | 32.5 | 39.9 | 0.2 | 0.0 |
| RELACS\_CTCF\_10000\_Rep2\_15min\_11 | 1.1 | 99.7 | 35.2 | 3.4 | 11.1 | 37.8 | 35.3 | 37.8 | 0.2 | 0.0 |
| RELACS\_CTCF\_10000\_Rep2\_15min\_12 | 0.9 | 99.3 | 35.3 | 3.4 | 10.6 | 38.2 | 35.5 | 38.2 | 0.6 | 0.0 |
| RELACS\_CTCF\_10000\_Rep2\_15min\_13 | 0.9 | 99.5 | 30.0 | 3.9 | 9.4 | 34.4 | 30.9 | 34.4 | 0.3 | 0.0 |
| RELACS\_CTCF\_10000\_Rep2\_15min\_14 | 0.7 | 99.6 | 31.2 | 3.3 | 9.6 | 35.9 | 32.0 | 35.9 | 0.3 | 0.0 |
| RELACS\_CTCF\_10000\_Rep2\_15min\_8 | 0.5 | 99.3 | 24.7 | 3.3 | 9.7 | 30.4 | 25.6 | 30.4 | 0.4 | 0.0 |
| RELACS\_CTCF\_10000\_Rep2\_15min\_9 | 1.0 | 99.7 | 34.8 | 3.4 | 10.4 | 38.1 | 35.2 | 38.1 | 0.2 | 0.0 |
| RELACS\_CTCF\_1000\_Rep2\_15min\_10 | 1.5 | 99.6 | 57.7 | 3.7 | 8.2 | 58.2 | 46.7 | 58.2 | 0.3 | 0.0 |
| RELACS\_CTCF\_1000\_Rep2\_15min\_11 | 1.0 | 99.6 | 54.9 | 3.7 | 8.2 | 55.7 | 51.8 | 55.7 | 0.3 | 0.0 |
| RELACS\_CTCF\_1000\_Rep2\_15min\_12 | 1.1 | 99.4 | 57.7 | 3.8 | 8.4 | 56.2 | 52.3 | 56.2 | 0.4 | 0.0 |
| RELACS\_CTCF\_1000\_Rep2\_15min\_13 | 1.0 | 99.5 | 49.0 | 4.0 | 8.1 | 51.5 | 46.3 | 51.5 | 0.4 | 0.0 |
| RELACS\_CTCF\_1000\_Rep2\_15min\_14 | 0.9 | 99.5 | 52.0 | 3.7 | 8.2 | 53.4 | 47.4 | 53.4 | 0.4 | 0.0 |
| RELACS\_CTCF\_1000\_Rep2\_15min\_8 | 0.6 | 99.2 | 42.9 | 3.7 | 8.8 | 46.6 | 39.0 | 46.6 | 0.5 | 0.0 |
| RELACS\_CTCF\_1000\_Rep2\_15min\_9 | 1.1 | 99.6 | 56.2 | 3.8 | 8.5 | 55.8 | 51.5 | 55.8 | 0.3 | 0.0 |
| RELACS\_CTCF\_100\_Rep2\_15min\_10 | 2.3 | 99.6 | 75.3 | 4.0 | 7.8 | 71.7 | 58.3 | 71.7 | 0.3 | 0.0 |
| RELACS\_CTCF\_100\_Rep2\_15min\_11 | 1.1 | 99.5 | 73.7 | 4.0 | 8.1 | 69.5 | 65.0 | 69.5 | 0.3 | 0.0 |
| RELACS\_CTCF\_100\_Rep2\_15min\_12 | 1.0 | 99.4 | 74.6 | 3.8 | 8.7 | 69.9 | 65.5 | 69.9 | 0.4 | 0.0 |
| RELACS\_CTCF\_100\_Rep2\_15min\_13 | 1.6 | 99.4 | 67.9 | 3.8 | 8.5 | 65.9 | 59.5 | 65.9 | 0.4 | 0.0 |
| RELACS\_CTCF\_100\_Rep2\_15min\_14 | 0.7 | 99.5 | 70.1 | 3.9 | 8.2 | 67.1 | 60.4 | 67.1 | 0.4 | 0.0 |
| RELACS\_CTCF\_100\_Rep2\_15min\_8 | 0.6 | 99.1 | 61.2 | 3.9 | 8.6 | 60.9 | 51.8 | 60.9 | 0.5 | 0.0 |
| RELACS\_CTCF\_100\_Rep2\_15min\_9 | 1.1 | 99.6 | 72.2 | 4.1 | 8.4 | 69.2 | 64.3 | 69.2 | 0.3 | 0.0 |
| RELACS\_H3K27me3\_10000\_Rep2\_15min\_10 | 5.6 | 99.3 | 9.2 | 5.1 | 11.5 | 8.0 | 5.1 | 8.0 | 0.6 | 0.0 |
| RELACS\_H3K27me3\_10000\_Rep2\_15min\_11 | 4.9 | 99.3 | 8.9 | 5.2 | 11.1 | 6.8 | 5.8 | 6.8 | 0.6 | 0.0 |
| RELACS\_H3K27me3\_10000\_Rep2\_15min\_12 | 4.1 | 99.3 | 8.5 | 5.0 | 10.5 | 6.8 | 5.9 | 6.8 | 0.5 | 0.0 |
| RELACS\_H3K27me3\_10000\_Rep2\_15min\_13 | 4.1 | 99.2 | 8.6 | 5.2 | 10.6 | 6.5 | 5.3 | 6.5 | 0.7 | 0.0 |
| RELACS\_H3K27me3\_10000\_Rep2\_15min\_14 | 3.5 | 99.3 | 8.6 | 5.2 | 10.7 | 6.4 | 5.1 | 6.4 | 0.6 | 0.0 |
| RELACS\_H3K27me3\_10000\_Rep2\_15min\_8 | 2.5 | 99.0 | 8.5 | 5.2 | 11.6 | 5.7 | 4.2 | 5.7 | 0.8 | 0.0 |
| RELACS\_H3K27me3\_10000\_Rep2\_15min\_9 | 4.3 | 99.4 | 8.7 | 5.1 | 10.9 | 6.7 | 5.7 | 6.7 | 0.5 | 0.0 |
| RELACS\_H3K27me3\_1000\_Rep2\_15min\_10 | 5.7 | 99.5 | 10.6 | 4.8 | 9.2 | 12.5 | 8.9 | 12.5 | 0.4 | 0.0 |
| RELACS\_H3K27me3\_1000\_Rep2\_15min\_11 | 4.4 | 99.5 | 10.5 | 4.8 | 9.2 | 11.5 | 10.1 | 11.5 | 0.4 | 0.0 |
| RELACS\_H3K27me3\_1000\_Rep2\_15min\_12 | 4.2 | 99.5 | 10.3 | 4.7 | 9.2 | 11.5 | 10.1 | 11.5 | 0.4 | 0.0 |
| RELACS\_H3K27me3\_1000\_Rep2\_15min\_13 | 3.6 | 99.3 | 10.0 | 5.0 | 9.4 | 10.5 | 8.8 | 10.5 | 0.5 | 0.0 |
| RELACS\_H3K27me3\_1000\_Rep2\_15min\_14 | 3.7 | 99.4 | 10.0 | 5.0 | 9.1 | 10.7 | 8.8 | 10.7 | 0.5 | 0.0 |
| RELACS\_H3K27me3\_1000\_Rep2\_15min\_8 | 2.6 | 99.1 | 9.4 | 5.0 | 9.7 | 9.4 | 7.1 | 9.4 | 0.6 | 0.0 |
| RELACS\_H3K27me3\_1000\_Rep2\_15min\_9 | 4.1 | 99.5 | 10.3 | 4.8 | 9.3 | 11.3 | 9.8 | 11.3 | 0.4 | 0.0 |
| RELACS\_H3K27me3\_100\_Rep2\_15min\_10 | 6.5 | 99.6 | 37.2 | 3.9 | 7.2 | 44.1 | 33.6 | 44.1 | 0.3 | 0.0 |
| RELACS\_H3K27me3\_100\_Rep2\_15min\_11 | 3.3 | 99.6 | 37.6 | 4.0 | 7.5 | 41.8 | 38.4 | 41.8 | 0.3 | 0.0 |
| RELACS\_H3K27me3\_100\_Rep2\_15min\_12 | 2.7 | 99.1 | 37.9 | 4.0 | 8.9 | 41.1 | 37.6 | 41.1 | 0.7 | 0.0 |
| RELACS\_H3K27me3\_100\_Rep2\_15min\_13 | 4.5 | 99.4 | 32.6 | 3.9 | 7.8 | 37.8 | 33.3 | 37.8 | 0.4 | 0.0 |
| RELACS\_H3K27me3\_100\_Rep2\_15min\_14 | 1.9 | 99.5 | 33.9 | 4.1 | 7.6 | 39.1 | 34.2 | 39.1 | 0.3 | 0.0 |
| RELACS\_H3K27me3\_100\_Rep2\_15min\_8 | 1.7 | 99.2 | 27.1 | 4.2 | 8.1 | 33.4 | 27.5 | 33.4 | 0.5 | 0.0 |
| RELACS\_H3K27me3\_100\_Rep2\_15min\_9 | 3.1 | 99.6 | 37.0 | 4.2 | 7.8 | 41.4 | 37.6 | 41.4 | 0.3 | 0.0 |
| RELACS\_H3K4me3\_10000\_Rep2\_15min\_10 | 2.7 | 99.4 | 7.9 | 1.9 | 9.9 | 12.1 | 7.9 | 12.1 | 0.4 | 0.0 |
| RELACS\_H3K4me3\_10000\_Rep2\_15min\_11 | 2.4 | 99.5 | 7.3 | 1.9 | 9.6 | 10.4 | 9.1 | 10.4 | 0.4 | 0.0 |
| RELACS\_H3K4me3\_10000\_Rep2\_15min\_12 | 2.1 | 99.2 | 7.1 | 2.0 | 9.5 | 10.1 | 8.8 | 10.1 | 0.6 | 0.0 |
| RELACS\_H3K4me3\_10000\_Rep2\_15min\_13 | 1.9 | 99.3 | 6.5 | 2.1 | 9.0 | 9.3 | 7.7 | 9.3 | 0.5 | 0.0 |
| RELACS\_H3K4me3\_10000\_Rep2\_15min\_14 | 1.7 | 99.4 | 6.4 | 1.9 | 9.1 | 9.5 | 7.7 | 9.5 | 0.5 | 0.0 |
| RELACS\_H3K4me3\_10000\_Rep2\_15min\_8 | 1.1 | 99.0 | 6.0 | 1.9 | 10.1 | 8.3 | 6.2 | 8.3 | 0.6 | 0.0 |
| RELACS\_H3K4me3\_10000\_Rep2\_15min\_9 | 2.1 | 99.5 | 7.0 | 1.9 | 9.5 | 10.3 | 8.8 | 10.3 | 0.4 | 0.0 |
| RELACS\_H3K4me3\_1000\_Rep2\_15min\_10 | 2.0 | 99.5 | 13.0 | 2.4 | 7.9 | 20.4 | 14.4 | 20.4 | 0.3 | 0.0 |
| RELACS\_H3K4me3\_1000\_Rep2\_15min\_11 | 1.7 | 99.6 | 13.0 | 2.5 | 8.0 | 19.0 | 17.0 | 19.0 | 0.3 | 0.0 |
| RELACS\_H3K4me3\_1000\_Rep2\_15min\_12 | 1.6 | 99.5 | 12.8 | 2.3 | 7.8 | 19.0 | 17.1 | 19.0 | 0.3 | 0.0 |
| RELACS\_H3K4me3\_1000\_Rep2\_15min\_13 | 1.3 | 99.4 | 11.3 | 2.7 | 7.9 | 16.8 | 14.4 | 16.8 | 0.4 | 0.0 |
| RELACS\_H3K4me3\_1000\_Rep2\_15min\_14 | 1.3 | 99.5 | 11.2 | 2.3 | 7.8 | 17.4 | 14.7 | 17.4 | 0.4 | 0.0 |
| RELACS\_H3K4me3\_1000\_Rep2\_15min\_8 | 0.8 | 99.1 | 9.5 | 2.3 | 8.3 | 14.8 | 11.7 | 14.8 | 0.5 | 0.0 |
| RELACS\_H3K4me3\_1000\_Rep2\_15min\_9 | 1.5 | 99.6 | 12.3 | 2.4 | 8.0 | 18.7 | 16.6 | 18.7 | 0.3 | 0.0 |
| RELACS\_H3K4me3\_100\_Rep2\_15min\_10 | 1.7 | 99.5 | 37.2 | 3.1 | 7.5 | 43.7 | 33.3 | 43.7 | 0.3 | 0.0 |
| RELACS\_H3K4me3\_100\_Rep2\_15min\_11 | 1.0 | 99.4 | 37.1 | 3.2 | 7.6 | 42.4 | 39.0 | 42.4 | 0.3 | 0.0 |
| RELACS\_H3K4me3\_100\_Rep2\_15min\_12 | 0.8 | 99.3 | 37.7 | 3.2 | 8.2 | 41.8 | 38.5 | 41.8 | 0.4 | 0.0 |
| RELACS\_H3K4me3\_100\_Rep2\_15min\_13 | 1.3 | 99.4 | 32.4 | 3.2 | 7.9 | 38.1 | 33.7 | 38.1 | 0.4 | 0.0 |
| RELACS\_H3K4me3\_100\_Rep2\_15min\_14 | 0.5 | 99.4 | 33.4 | 3.3 | 7.6 | 39.2 | 34.6 | 39.2 | 0.3 | 0.0 |
| RELACS\_H3K4me3\_100\_Rep2\_15min\_8 | 0.4 | 98.5 | 27.0 | 3.3 | 8.3 | 33.8 | 28.2 | 33.8 | 0.8 | 0.0 |
| RELACS\_H3K4me3\_100\_Rep2\_15min\_9 | 0.8 | 99.5 | 36.9 | 3.2 | 7.9 | 41.6 | 37.9 | 41.6 | 0.3 | 0.0 |
| RELACS\_Input\_10000\_Rep2\_15min\_10 | 2.7 | 99.6 | 6.4 | 3.1 | 10.0 | 6.6 | 4.7 | 6.6 | 0.3 | 0.0 |
| RELACS\_Input\_10000\_Rep2\_15min\_11 | 2.1 | 99.6 | 6.1 | 3.0 | 10.1 | 5.6 | 4.9 | 5.6 | 0.3 | 0.0 |
| RELACS\_Input\_10000\_Rep2\_15min\_12 | 2.2 | 99.5 | 6.0 | 3.0 | 9.7 | 5.7 | 5.1 | 5.7 | 0.4 | 0.0 |
| RELACS\_Input\_10000\_Rep2\_15min\_13 | 1.9 | 99.5 | 5.8 | 3.1 | 9.1 | 5.5 | 4.6 | 5.5 | 0.4 | 0.0 |
| RELACS\_Input\_10000\_Rep2\_15min\_14 | 1.7 | 99.5 | 5.7 | 3.1 | 9.3 | 5.3 | 4.3 | 5.3 | 0.3 | 0.0 |
| RELACS\_Input\_10000\_Rep2\_15min\_8 | 1.2 | 99.3 | 5.5 | 3.1 | 9.3 | 4.8 | 3.7 | 4.8 | 0.4 | 0.0 |
| RELACS\_Input\_10000\_Rep2\_15min\_9 | 2.1 | 99.6 | 6.0 | 3.1 | 9.8 | 5.6 | 4.9 | 5.6 | 0.3 | 0.0 |
| RELACS\_Input\_1000\_Rep2\_15min\_10 | 3.9 | 99.6 | 6.2 | 3.3 | 8.2 | 6.9 | 5.2 | 6.9 | 0.2 | 0.0 |
| RELACS\_Input\_1000\_Rep2\_15min\_11 | 3.2 | 99.6 | 6.3 | 3.3 | 8.4 | 6.9 | 5.9 | 6.9 | 0.2 | 0.0 |
| RELACS\_Input\_1000\_Rep2\_15min\_12 | 3.1 | 99.6 | 6.0 | 3.2 | 8.3 | 6.4 | 5.5 | 6.4 | 0.2 | 0.0 |
| RELACS\_Input\_1000\_Rep2\_15min\_13 | 2.7 | 99.4 | 6.1 | 3.5 | 8.1 | 6.2 | 5.0 | 6.2 | 0.3 | 0.0 |
| RELACS\_Input\_1000\_Rep2\_15min\_14 | 2.9 | 99.5 | 6.0 | 3.4 | 7.9 | 6.3 | 5.0 | 6.3 | 0.3 | 0.0 |
| RELACS\_Input\_1000\_Rep2\_15min\_8 | 2.1 | 99.2 | 5.7 | 3.3 | 8.0 | 5.7 | 4.2 | 5.7 | 0.4 | 0.0 |
| RELACS\_Input\_1000\_Rep2\_15min\_9 | 3.4 | 99.6 | 6.3 | 3.3 | 8.2 | 6.8 | 5.8 | 6.8 | 0.2 | 0.0 |
| RELACS\_Input\_100\_Rep2\_15min\_10 | 3.5 | 99.2 | 6.3 | 3.2 | 8.5 | 7.3 | 4.7 | 7.3 | 0.4 | 0.0 |
| RELACS\_Input\_100\_Rep2\_15min\_11 | 1.9 | 98.9 | 6.6 | 3.3 | 9.2 | 6.8 | 5.6 | 6.8 | 0.4 | 0.0 |
| RELACS\_Input\_100\_Rep2\_15min\_12 | 1.7 | 98.8 | 6.4 | 3.2 | 9.6 | 6.4 | 5.2 | 6.4 | 0.4 | 0.0 |
| RELACS\_Input\_100\_Rep2\_15min\_13 | 2.8 | 99.0 | 6.3 | 3.3 | 8.7 | 6.6 | 5.1 | 6.6 | 0.4 | 0.0 |
| RELACS\_Input\_100\_Rep2\_15min\_14 | 1.3 | 98.8 | 6.2 | 3.4 | 8.8 | 6.2 | 4.7 | 6.2 | 0.4 | 0.0 |
| RELACS\_Input\_100\_Rep2\_15min\_8 | 1.2 | 98.4 | 6.0 | 3.5 | 8.4 | 5.9 | 4.2 | 5.9 | 0.7 | 0.0 |
| RELACS\_Input\_100\_Rep2\_15min\_9 | 1.9 | 98.6 | 6.5 | 3.4 | 9.1 | 6.5 | 5.2 | 6.5 | 0.4 | 0.0 |

×

#### Table Rsyz: Columns

Uncheck the tick box to hide columns. Click and drag the handle on the left to change order.

Show All
Show None

| Sort | Visible | Group | Column | Description | ID | Scale |
| --- | --- | --- | --- | --- | --- | --- |
| || |  | deepTools bamPEFragmentSize | M entries | Number of entries in the file (millions) | `M Entries` | None |
| || |  | deepTools bamPEFragmentSize | % Aligned | Percent of aligned entries | `% Aligned` | None |
| || |  | deepTools bamPEFragmentSize | % Tot. Filtered | Percent of alignment that would be filtered for any reason. | `% Filtered` | None |
| || |  | deepTools bamPEFragmentSize | % Blacklisted | Percent of alignments falling (at least partially) inside a blacklisted region | `% Blacklisted` | None |
| || |  | deepTools bamPEFragmentSize | % Missing Flags | Percent of alignments lacking at least on flag specified by --samFlagInclude | `% Missing Flags` | None |
| || |  | deepTools bamPEFragmentSize | % Forbidden Flags | Percent of alignments having at least one flag specified by --samFlagExclude | `% Forbidden Flags` | None |
| || |  | deepTools bamPEFragmentSize | % deepTools Dupes | Percent of alignments marked by deepTools as being duplicates | `% deepTools Dupes` | None |
| || |  | deepTools bamPEFragmentSize | % Duplication | Percent of alignments originally marked as being duplicates | `% Duplication` | None |
| || |  | deepTools bamPEFragmentSize | % Singletons | Percent of alignments that are singletons (i.e., paired-end reads where the mates don't align as a pair | `% Singletons` | None |
| || |  | deepTools bamPEFragmentSize | % Strand Filtered | Percent of alignments arising from the wrong strand | `% Strand Filtered` | None |

Close

---

## Bowtie 2

Bowtie 2 is an ultrafast and memory-efficient tool for aligning sequencing reads to long reference sequences.

Help

This plot shows the number of reads aligning to the reference in different ways.  
*Please note that single mate alignment counts are halved to tally with pair counts properly.*

There are 6 possible types of alignment:
 ***PE mapped uniquely**: Pair has only one occurence in the reference genome.* **PE mapped discordantly uniquely**: Pair has only one occurence but not in proper pair.
 ***PE one mate mapped uniquely**: One read of a pair has one occurence.* **PE multimapped**: Pair has multiple occurence.
 ***PE one mate multimapped**: One read of a pair has multiple occurence.* **PE neither mate aligned**: Pair has no occurence.

Number of Reads
Percentages

Created with Highcharts 5.0.6# ReadsChart context menuExport PlotBowtie 2: PE Alignment ScoresPE mapped uniquelyPE mapped discordantly uniquelyPE one mate mapped uniquelyPE multimappedPE one mate multimappedPE neither mate alignedRELACS\_CTCF\_10000\_Rep2\_15min\_10.Bowtie2RELACS\_CTCF\_10000\_Rep2\_15min\_14.Bowtie2RELACS\_CTCF\_1000\_Rep2\_15min\_11.Bowtie2RELACS\_CTCF\_1000\_Rep2\_15min\_8.Bowtie2RELACS\_CTCF\_100\_Rep2\_15min\_12.Bowtie2RELACS\_CTCF\_100\_Rep2\_15min\_9.Bowtie2RELACS\_H3K27me3\_10000\_Rep2\_15min\_13.Bowtie2RELACS\_H3K27me3\_1000\_Rep2\_15min\_10.Bowtie2RELACS\_H3K27me3\_1000\_Rep2\_15min\_14.Bowtie2RELACS\_H3K27me3\_100\_Rep2\_15min\_11.Bowtie2RELACS\_H3K27me3\_100\_Rep2\_15min\_8.Bowtie2RELACS\_H3K4me3\_10000\_Rep2\_15min\_12.Bowtie2RELACS\_H3K4me3\_10000\_Rep2\_15min\_9.Bowtie2RELACS\_H3K4me3\_1000\_Rep2\_15min\_13.Bowtie2RELACS\_H3K4me3\_100\_Rep2\_15min\_10.Bowtie2RELACS\_H3K4me3\_100\_Rep2\_15min\_14.Bowtie2RELACS\_Input\_10000\_Rep2\_15min\_11.Bowtie2RELACS\_Input\_10000\_Rep2\_15min\_8.Bowtie2RELACS\_Input\_1000\_Rep2\_15min\_12.Bowtie2RELACS\_Input\_1000\_Rep2\_15min\_9.Bowtie2RELACS\_Input\_100\_Rep2\_15min\_13.Bowtie20500k1000k1500k2000k2500k3000k3500kCreated with MultiQC

| RELACS\_CTCF\_10000\_Rep2\_15min\_10.Bowtie2 | | |
| --- | --- | --- |
| PE mapped uniquely: | 395157 | (62.9%) |
| PE mapped discordantly uniquely: | 36886 | (5.9%) |
| PE one mate mapped uniquely: | 10261 | (1.6%) |
| PE multimapped: | 164591 | (26.2%) |
| PE one mate multimapped: | 19490 | (3.1%) |
| PE neither mate aligned: | 2223 | (0.4%) |

**MultiQC v1.3**
- Written by Phil Ewels,
available on GitHub.

This report uses HighCharts,
jQuery,
jQuery UI,
Bootstrap,
FileSaver.js and
clipboard.js.

×

### Plot Table Data

Select Column

Select Column

Please select two table columns.

Close

×

### Regex Help

Toolbox search strings can behave as regular expressions
(regexes). Click a button below to see an example of it in action. Try
modifying them yourself in the text box.

`^` (start of string)
`$` (end of string)
`[]` (character choice)
`\d` (shorthand for `[0-9]`)
`\w` (shorthand for `[0-9a-zA-Z_]`)
`.` (any character)
`\.` (literal full stop)
`()` `|` (group / separator)
`*` (prev char 0 or more)
`+` (prev char 1 or more)
`?` (prev char 0 or 1)
`{}` (char num times)
`{,}` (count range)

```
samp_1
samp_1_edited
samp_2
samp_2_edited
samp_3
samp_3_edited
prepended_samp_1
tmp_samp_1_edited
tmpp_samp_1_edited
tmppp_samp_1_edited
#samp_1_edited.tmp
samp_11
samp_11111
```

See regex101.com for a more heavy duty testing suite.

Close
